# Unexpected gender differences in progressive supranuclear palsy reveal efficacy for davunetide in women

**DOI:** 10.1038/s41398-023-02618-9

**Published:** 2023-10-16

**Authors:** Illana Gozes, Guy Shapira, Alexandra Lobyntseva, Noam Shomron

**Affiliations:** 1 Elton Laboratory for Molecular Neuroendocrinology, Department of Human Molecular Genetics and Biochemistry, Faculty of Medicine, Adams Super Center for Brain Studies and Sagol School of Neuroscience, Tel Aviv University, 69978 Tel Aviv, Israel; 2 Department of Cell and Developmental Biology, Faculty of Medicine, Sagol School of Neuroscience, Edmond J Safra Center for Bioinformatics, Tel Aviv University, 69978 Tel Aviv, Israel

**Keywords:** Diseases, Pathogenesis

## Abstract

Progressive supranuclear palsy (PSP) is a pure tauopathy, implicating davunetide, enhancing Tau-microtubule interaction, as an ideal drug candidate. However, pooling patient data irrespective of sex concluded no efficacy. Here, analyzing sex-dependency in a 52 week-long- PSP clinical trial (involving over 200 patients) demonstrated clear baseline differences in brain ventricular volumes, a secondary endpoint. Dramatic baseline ventricular volume-dependent/volume increase correlations were observed in 52-week-placebo-treated females (*r* = 0.74, *P* = 2.36^–9^), whereas davunetide-treated females (like males) revealed no such effects. Assessment of primary endpoints, by the PSP Rating Scale (PSPRS) and markedly more so by the Schwab and England Activities of Daily Living (SEADL) scale, showed significantly faster deterioration in females, starting at trial week 13 (*P* = 0.01, and correlating with most other endpoints by week 52). Twice daily davunetide treatments slowed female disease progression and revealed significant protection according to the SEADL scale as early as at 39 weeks (*P* = 0.008), as well as protection of the bulbar and limb motor domains considered by the PSPRS, including speaking and swallowing difficulties caused by brain damage, and deterioration of fine motor skills, respectably (*P* = 0.01), at 52 weeks. Furthermore, at 52 weeks of trial, the exploratory Geriatric Depression Scale (GDS) significantly correlated with the SEADL scale deterioration in the female placebo group and demonstrated davunetide-mediated protection of females. Female-specific davunetide-mediated protection of ventricular volume corresponded to clinical efficacy. Together with the significantly slower disease progression seen in men, the results reveal sex-based drug efficacy differences, demonstrating the neuroprotective and disease-modifying impact of davunetide treatment for female PSP patients.

## Introduction

Davunetide [also known as AL-108, C201, NAP and NAPVSIPQ (single amino acid letter code)] is the smallest neuroprotective peptide derived from activity-dependent neuroprotective protein (ADNP) [1]. Davunetide and pipeline products protect nerve cells by associating with microtubule end-binding proteins (EB1/EB3) via the SxIP motif in NAPVSIPQ, thus enhancing microtubule dynamics and Tau-microtubule interactions, in turn, protecting the synapse [2–5]. We have recently extended davunetide target specificity, showing a Src homology 3 (SH3) domain–ligand association site in NAP (NAPVISP) as being responsible for controlling signaling pathways regulating the cytoskeleton, including actin–Tau interactions [6]. The NAP ADNP motif, furthermore, binds to the armadillo domain of beta-catenin [7], important for ADNP neurodevelopmental/maintenance functions [8], and linked with Alzheimer’s disease (AD) amyloid/Tau pathology [9].

Concentrating on the NAP (davunetide) parent protein ADNP (which regulates steroid biosynthesis genes [10]), we discovered extensive sex-dependent deficiencies in ADNP-mutated mice, coupled with NAP corrective effects [5, 11]. For example, we showed slower axonal transport in the mouse olfactory nerve due to ADNP deficiency, a phenomenon accentuated in males and corrected by application of the NAP EB1/EB3-interacting moiety SKIP [2]. We further showed mouse sex-dependent changes in tubulin isotype expression associated with modified ADNP levels and brain maturation [2]. In turn, we found NAP (davunetide) regulation of tubulin isotype expression toward neuronal/brain maturation [12]. Additionally, we found higher *ADNP* mRNA transcript levels in post-mortem male hippocampi, as compared with those from females [13]. Corroborating studies evaluating traumatic axonal injury in human and rodent neurons relying on ultra-structural analysis revealed that female axons were consistently smaller, with fewer microtubules, relative to male axons. Computational modeling indicated that these differences place microtubules in female axons at a greater risk for failure during trauma, leading to neurodegeneration [14]. Still, previous clinical trials with davunetide did not separately address women and men. Regardless, davunetide treatment resulted in cognitive score enhancement in amnestic mild cognitive impairment (aMCI), preceding AD [15] and protection of daily living activities in schizophrenia patients [15, 16]. The choice of aMCI as a therapeutic target for davunetide is obvious, as the drug candidate protects against tauopathy [3, 5, 17–19] and cell death [1, 20], with aMCI being the precursor of AD, the major tauopathy. The choice of cognitive impairments (reflected in aberrant daily activities) in schizophrenia is equally relevant, as by enhancing microtubule dynamics, davunetide augments the autophagy process, which is deficient in schizophrenia [21, 22]. Importantly, ADNP regulates autophagy, interacting with autophagy-controlling proteins, such as microtubule-associated protein 1 light chain 3 (LC3) [23], and protecting beclin 1 expression in a sex-dependent manner [2]. At the same time, NAP (davunetide) enhances ADNP-LC3 interaction and beclin 1 expression, thus providing neuroprotection [2, 21, 23–26].

Given that tauopathy is a major outcome of ADNP deficiency or pathological *ADNP* mutations in mice [5, 17] and humans [10], and that NAP (davunetide) offered protection against tauopathy in multiple pre-clinical models [5, 17, 27], as well as provided indications of encouraging increased cognitive scores in an aMCI trial [15], progressive supranuclear palsy (PSP) was deemed to be an ideal target for davunetide-mediated protection. However, the original PSP clinical study in which the entire tested population was addressed, namely, where male and female patient data were pooled, showed the safety of the drug candidate but did not meet its endpoints [28]. In trying to understand these results, one explanation considered was the possibility of dosage beyond the hormetic (inverted U-shaped) dose-response curve [29], identified in pre-clinical studies [1], and further suggested in clinical trials. For instance, in the case of schizophrenia, a 5 mg daily dose was significantly active, yet a higher 15 mg twice daily dose led to results neither significantly different from those obtained with the 5 mg daily dose, nor as obtained with the placebo, suggestive of plateauing towards a bell-shaped response, even though the study was under-powered [16]. In aMCI that precedes AD, there was dose dependency (as revealed upon administering the above doses) [30, 31], suggesting disease specificity. Conversely, the dosage used with PSP (30 mg, twice daily) was two-fold higher than the highest dosages used with aMCI and schizophrenia [16, 28], possibly reflecting how a bell-shaped response had been reached.

Closer examination of Tau may provide an alternative explanation for the trial results. Indeed, understanding the regulation of Tau (*MAPT*) mRNA splicing is important for defining the etiology of PSP, as well as of other tauopathies. Tauopathies can be categorized by the presence of Tau aggregates containing 3 (3R) and/or 4 (4R) microtubule-binding domain repeats (determined by inclusion/exclusion of *MAPT* exon 10) and by the inclusion/exclusion of exons 2 and 3, that translate into two N-terminal Tau domains. In PSP, there is an accumulation of Tau 1N4R isoforms, whereas in the AD temporal cortex, the 0N isoform predominates [32]. Importantly, davunetide/ADNP enhances Tau-microtubule association [4]. In turn, ADNP directly interacts with the splicing machinery, possibly to suppress *MAPT* exon 10 inclusion [33]. Furthermore, while the davunetide trial described above addressed 4R tauopathy PSP, potentially representing an ideal target population for davunetide-mediated protection, the results were deemed negative [28]. Part of this negative result was attributed to preferential NAP-mediated enhancement of dynamic 3R Tau (i.e., Tau containing three microtubule interaction sites), as opposed to the 4R-based Tau-microtubule interaction [4]. Yet, given how alternative splicing may differ between males and females, separating the results according to patient sex could be of interest [34].

Lastly, in trying to understand what drives PSP tauopathy, an elegant study identified genetic variations and increases in filamin A in PSP brains. Accordingly, increased filamin A levels enhanced the phosphorylation and insolubility of Tau through interactions with actin filaments. In addition, reduced filamin A levels corrected aberrant Tau levels in cells cultured from PSP patients, while transgenic mice carrying human filamin A recapitulated Tau pathology in neurons [35]. In this respect, we have shown that ADNP contains an actin-binding domain and that NAP corrects improper actin-associated protein interactions [6]. At the same time, a filamin A fragment localizes to the nucleus to regulate androgen receptor and co-activator functions, thus implicating sexual dichotomy and the necessity to analyze males and females separately [36].

Gender differences in PSP were not appreciated when the results of the earlier davunetide-based clinical trial were evaluated [28]. Such re-evaluation is warranted, given the results of a recent study addressing gender differences in the phenotypic expression and progression of PSP by reviewing medical records of patients diagnosed with PSP over a 21-year period. This latter study evaluated the interval between disease onset and the presentation of the five clinical disability milestones of PSP, namely, wheelchair dependency, unintelligible speech, severe dysphagia, severe cognitive impairment, and urinary catheterization, to determine disease progression in 334 patients (209 males and 125 females). The study indicated that the males were older than the females at disease onset and showed a longer duration of illness at the time of presentation. Tremors were more common, the PSP-Parkinsonism phenotype was more frequent, and the time until wheelchair dependency was earlier in males. In contrast, falls within a year of disease onset, apathy, and executive dysfunction were more frequent in females and the time until unintelligible speech, severe dysphagia, and cognitive impairment were also earlier [37]. Thus, gender differences exist in PSP in terms of clinical characteristics and disease progression, in agreement with the proposed molecular mechanism of the disease.

Taken together with ADNP-mediated promotion of sex-dependent neuronal morphogenesis/plasticity [38], ADNP-mediated regulation of steroid biosynthesis genes [10], sexually dichotomized ADNP expression levels [13], for example regulated by estrous cycle [39], gonadotropin-releasing hormone receptor (*Gnrhr*)-correlated ADNP expression [40], estrogen-mediated regulation of alternative splicing [34], estrogen level-/menopause-mediated association with Tau deposition [41], ADNP binding to actin, and NAP (davunetide)-mediated enhancement of actin–Tau interactions [6], all imply the existence of gender-based differences in PSP patient responses to the ADNP-derived drug candidate davunetide. The relevant data were thus re-evaluated and gender-based differences in PSP patient responses to davunetide were substantiated here for the first time. Specifically, the results showed that davunetide afforded significant protection to women suffering from PSP.

## Methods

### Data source

We (I. Gozes) [1] invented davunetide and co-founded Allon Therapeutics with a license for clinical development. Upon closure, Allon Therapeutics-funded PSP clinical results (Clinical Study Report) were transferred to us, allowing further data mining. Here, we have extensively re-analyzed these data separating males and females.

In a double-blind, parallel group phase 2/3 trial [28], participants were randomly assigned with permuted blocks in a 1:1 ratio to davunetide (NAP, 30 mg twice daily, intranasally) or placebo for 52 weeks at 48 centers in Australia, Canada, France, Germany, the UK, and the USA. Ethics approval was obtained at each site from the local ethics committee and all participants gave written informed consent at the recruitment visit as per local regulations. Study design is partly delineated in Fig. S1 (adapted from the original clinical trial description, which includes a detailed study profile [28]). Participants met the modified Neuroprotection and Natural History in Parkinson Plus Syndrome study criteria for PSP (inclusion criteria). Exclusion criteria included a clear and robust benefit from levodopa at the time of screening, evidence of motor neuron disease, or use of putative disease-modifying drugs for PSP [28]. Endpoints were originally selected to depict davunetide drug safety and efficacy in relation to disease progression [28]. Primary endpoints were the change from baseline on the PSP Rating Scale (PSPRS, 28 items, 6 categories, 0 = normal, 100 = most disability) and Schwab and England Activities of Daily Living (SEADL) scale (11-point rating scale, 0 = vegetative, 100% = completely independent, 10% increments). Data were collected at weeks 0, 6, 13, 26, 39, and 52 (Fig. S1). A secondary endpoint was brain ventricular volume change (including the lateral ventricles, the temporal horn of the lateral ventricles, and the third ventricle [42]), as measured in boundary shift integral analysis of T1-weighted magnetic resonance imaging (MRI) scans, mostly collected at weeks 0 and 52. An additional secondary endpoint included Clinical Global Impression of Change (CGIC). Exploratory endpoints included Clinical Global Impression of Disease Severity (CGIds), Geriatric Depression scale (GDS), Repeatable Battery for the Assessment of Neuropsychological Disease Severity (RBANS), phonemic fluency (words per min), color trail2 (assessing sustained attention in adults) and letter number sequence (short-term memory skills), all comprehensively measuring disease severity and progression, as originally introduced [28]. All participants and study personnel were masked to treatment assignment. The trial was registered with clinicaltrials.gov (number NCT01110720). The original article maintained that 313 participants were randomly assigned to davunetide (*n* = 157) or placebo (*n* = 156) groups. Of these, 241 (77%) completed the study (118 and 123 in the davunetide and placebo groups, respectively) [28]. The clinical study report transferred to us included 312 subjects, some with most or all data, others with very little data. Our analysis ended up using only the subjects with complete data, since the broader cohort produced largely similar results, without adding much significance or statistical strength. Thus, the data presented below encompassed all the available patients at 52 weeks of trial, almost equally distributed between males and females, and davunetide and placebo groups (see legends to the figures). Tables S1 and S2 include only participants for whom data was available for all measurements. As delineated in the first study article, an intention-to-treat analysis was used for the primary and secondary outcomes that included all participants who received at least one dose of medication and had a post-baseline efficacy assessment [28].

To understand sex/age/baseline differences in disease progression and drug influence, we re-evaluated the study results. Details are given in the original study publication [28] coupled with an outline in the “Results (Data source).” In addition, we individually assessed each of the six disability domains of the primary PSPRS endpoint, namely, [1] daily activities (by history, seven items with a total maximum score of 24), [2] behavior (mentation, emotional, interference with activity of daily living, maximum score of 16), [3] bulbar (speech and swallowing, maximal score of 8), [4] ocular motor (eye and eyelid activity, 16 points), [5] limb motor (fine motor skills including tremor, 16 points), and [6] gait/midline (gross motor skills, 20 points) (see https://www.physio-pedia.com/Progressive_Supranuclear_Palsy_Rating_Scale_(PSP-RS)). The Supplementary File also includes CSF measurements performed as described [43] on a very small subset of patients (“Results”).

### Statistical considerations

Unless otherwise stated, statistical tests and visualizations were performed in R, version 4.2.1 (2022-06-23) with the base libraries, rstatix 0.7.2 [44], ggplot2, and ggplot2 extension packages. All correlation statistics were computed using the standard Pearson method for linear regression, depicting the Pearson correlation coefficient *r*. We define an alpha value of 0.05 as our threshold for significance. The values used to calculate correlations and time-series statistics were normalized according to the respective baseline levels (percent difference from baseline value). Pairwise comparisons were made using the Wilcoxon signed-rank test.

Analyses using GraphPad Prism 7.0 software included D’Agostino and Pearson test for normality coupled with log10 transformation to achieve normal distribution. Two-way analysis of variance followed by the Sidak multiple comparisons test was further used to assess significant differences.

## Results

### Significant sex-based baseline differences

**Fig. 1:**
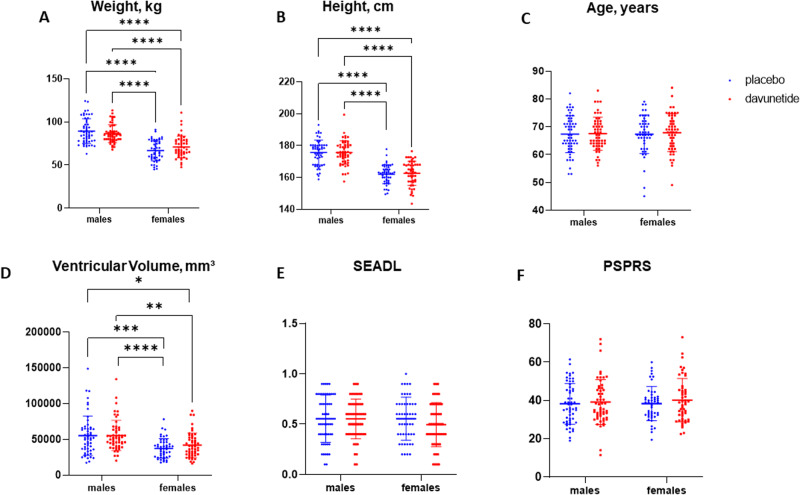
Significant baseline sex differences in brain ventricular volumes. The baseline demographics of the study cohorts are shown, namely, weight (**A**) and height (**B**), age (**C**), ventricular volumes (**D**), SEADL scale (**E**) and PSPRS (**F**). Placebo-treated subjects are in blue and davunetide-treated subjects are in red, with males (*n* = 55 placebo, *n* = 55 davunetide) and females (*n* = 48 placebo, *n* = 51 davunetide) being clearly separated. Means ± S.D. are denoted on the graphs. The GraphPad Prism 7.0 software was used to analyze the results. Following D’Agostino and Pearson normality distribution test, the datasets presented on (**A**, **B**, **D**, **F**) appeared to be lognormal distributed. Thus, for these data sets, log10 transformations were performed and were further analyzed by two-way ANOVA followed by the Sidak multiple comparisons test. Original data was plotted. For (**C**, **E**) data sets, normal distribution was found, hence, two-way ANOVA followed by Sidak multiple comparisons test was performed directly. Statistically significant differences are denoted as follows: *P < 0.05; **P < 0.01; ***P < 0.001; **** *P* < 0.0001.

Unlike the original analysis [28], we here re-analyzed the same data for females and males separately, starting from baseline characteristics. Figure 1 reveals that at baseline, the tested population differed by weight and height, with obviously higher male values. Furthermore, on average, the randomized davunetide-treated female group showed significantly increased weight, as compared to the placebo-treated group. There were no differences in age distribution among all tested study groups. While the primary endpoints, the PSPRS and SEADL scale baseline measures, did not differ between males and females, high variability was noted per sex.

For the secondary endpoint CGIC, measurements started at week 6, with the results showing similarity for all tested groups (average 4.1–4.3 out of 7 points). Concentrating on brain structure, and as expected from previous evaluations [45], MRI measures of ventricular volumes (a secondary endpoint) were larger in males at baseline, as compared to the tested females (Fig. 1). This suggests that analysis of the results according to sex is required in a neuroprotective drug trial, regardless of the original design.

### Davunetide treatment provides neuroprotection against brain ventricular volume-dependent increases in female PSP patients

**Fig. 2:**
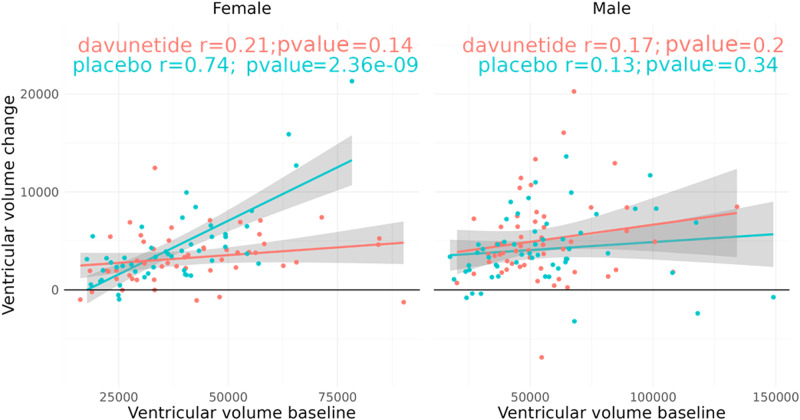
Davunetide treatment provides significant neuroprotection against baseline brain ventricular volume-dependent increases in female PSP patients. Linear regressions (specifically, the Pearson correlation coefficient *r*) correlating absolute ventricular volume change (mm^3^) with baseline volume (mm^3^), as measured by MRI after 52 weeks of trial, are presented. Males (*n* = 55 placebo, *n* = 55 davunetide) and females (*n* = 48 placebo, *n* = 51 davunetide) are separately pictured. See the main text (“Data source”) and “Methods” for further details.

Given the significant sex differences in ventricular volumes, with davunetide treatment providing neuroprotection in pre-clinical studies [1, 5, 11, 20, 46], we initially focused on the percent of baseline ventricular volume change at 52 weeks (MRI data), assessing any davunetide-mediated protective effects in PSP patients. Separating the study population by sex and baseline ventricular volume measures revealed dramatic placebo group increases correlating with the baseline ventricular volumes (*r* = 0.74; *P* = 23.6^–9^) in the female cohort, with complete protection being provided by twice daily intranasal davunetide application (Fig. 2). Unlike what was seen with females, the male cohorts showed no dependence on baseline ventricular volumetric measures nor any drug-linked effects after 52 weeks of treatment.

Similar results were obtained when the entire population was divided by sex (Fig. S2; individual level, Fig. S3). Exploratory measurements considering a limited number of available women also showed similar trends in total Tau and phosphorylated Tau level reductions in the cerebrospinal fluid (CSF) of 52 week davunetide-treated patients, as compared to those treated with the placebo (Fig. S4) [47].

### Sex-specific correlations of the study demographics and endpoints

**Fig. 3:**
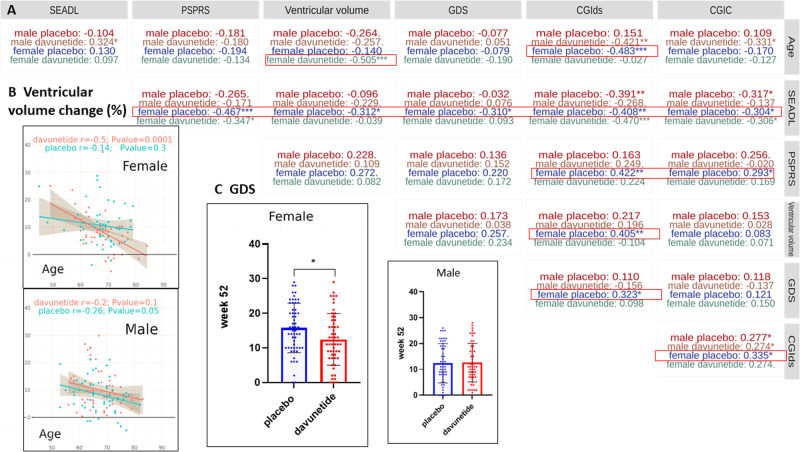
Substantial sex differences indicate that davunetide-mediated neuroprotection is significantly correlated with multiple study endpoints and age. **A** Shown are the Pearson correlation coefficient *r* values of plots reflecting percent change relative to baseline after 52 weeks of davunetide or placebo treatment, including the study primary endpoints (PSPRS and SEADL scale), the secondary endpoints of brain ventricular volume change and CGIC, the exploratory endpoints CGIds and CGD, as well as the demographic characteristics of age. Color-coded significance is denoted by *P < 0.05; **P < 0.01; ***P < 0.001. “.” denotes 0.05 < *P* < 0.1. Further variables including precise “*n*” for each variable and additional endpoint comparisons are delineated in Table S1, further correlations are shown in Table S2. **B** Linear regressions (depicting the Pearson correlation coefficient *r*) separating males and females according to davunetide or placebo treatment as measured by MRI are shown. The percent baseline change in ventricular volume at 52 weeks of treatment, correlating to age is presented. **C** Geriatric Depression Scale (GDS) values depicting the change from baseline of the overall scores in males (*n* = 52, placebo; *n* = 53, davunetide) and females (*n* = 57, placebo; *n* = 54, davunetide), as visualized by GraphPad Prism 7.0 software followed by a Wilcoxon test (Table S1).

Figure 3A depicts correlations of percent change compared to baseline after 52 weeks of davunetide or placebo treatment, including the study primary endpoints of the PSPRS and SEADL scale, the secondary endpoints of brain ventricular volume change and CGIC, and the exploratory endpoints GDS and CGIds. Demographic characteristics, such as weight and height (Fig. 1), did not correlate with the study primary endpoints, nor with brain ventricular volume percent increase after 52 weeks of treatment (e.g., *r* = 0.04 and 0.05, respectively, for the female davunetide-treated group).

In contrast, the percent change in ventricular volume over the 52-week trial period significantly correlated with the demographic parameter of age only in davunetide-treated females (*r* = −0.505***, Fig. 3A). Thus, while the placebo group did not show any difference in the percentage of ventricular volume increase during the trial period (52 weeks) across age groups, the davunetide-treated group exhibited a significant neuroprotective effect in women older than 60 years of age (Fig. 3B). Male placebo and davunetide groups behaved similarly in terms of ventricular volume changes across different ages (Fig. 3B).

Female placebo group-associated neurodegeneration, reflected by the increase in brain ventricular volume, was directly correlated with the primary endpoints of the trial, i.e., SEADL scale (*r* = −0.312*) and PSPRS (almost significant, *r* = 0.272.), as well as with the exploratory CGIds (*r* = 0.405**), protection was seen in the davunetide-treated cohort (i.e., almost no correlation). Furthermore, the CGIds percent change correlated with age and was highest in the youngest placebo-treated women (*r* = −0.483***, Fig. 3A). Strikingly, the SEADL scale and CGIds measures in the female placebo cohort significantly correlated with all other endpoints (red boxes). Furthermore, the SEADL scale and PSPRS percent changes from baseline to 52 treatment weeks were significantly correlated only in the female groups (*r* = −0.347*, davunetide; *r* = −0.467**, placebo). The male disease course differed from that of females in that the only significant correlations seen in the placebo male group were with SEADL scale, CGIC and CGIds. Furthermore, in males, the CGIC and CGIds measures similarly correlated in the placebo and davunetide cohorts (Fig. 3A). This is indicative of a female-specific disease course coupled to davunetide-mediated benefit (Fig. 3A, B).

The exploratory GDS score (measuring geriatric depression) showed correlation only in the female placebo group, significantly doing so with CGIds and SEADL scale (*r* = 0.323*, −0.310*, respectively) and almost significantly so with ventricular volume (*r* = 0.257). Wilcoxon testing (after 52 weeks of trial) further indicated a highly significant sex difference in the placebo groups (**P = 0.00867, Table S1), with the GDS score improving significantly in davunetide-treated females, as compared to the placebo group, at week 52 (*P = 0.0178, Fig. 3C and Table S1). Similar comparisons of the RBANS measurements (Table S1) showed a significant positive davunetide-mediated effect on expressive language functioning (language) in females alone (*P = 0.0107), and a significant davunetide-placebo difference in visuospatial constructional (namely, the ability to copy a design from a model) only in males, further highlighting sex differences. This was extended to a correlation table of all study endpoints (Table S2), highlighting significant correlations between the different RBANS measures.

### Significantly faster disease progression is seen in females

**Fig. 4:**
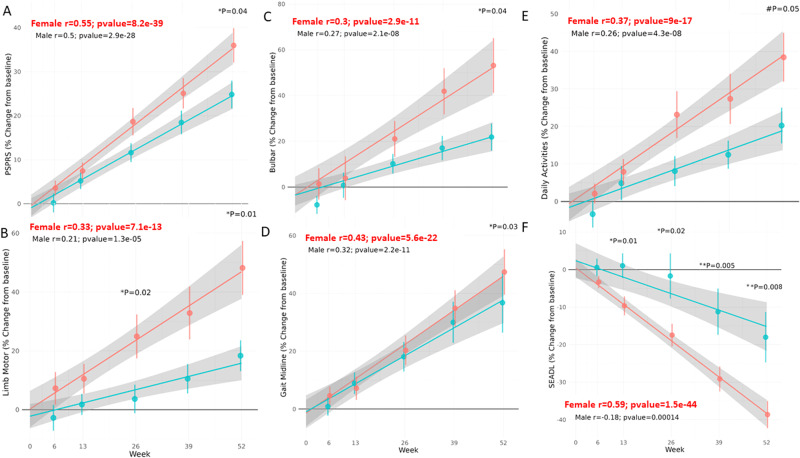
PSP develops significantly faster in females, as shown for SEADL scale and PSPRS measures, and is further accentuated in multiple PSPRS domains. Linear regressions (depicting the Pearson correlation coefficient *r*) separating placebo-treated males and females, measuring percent change relative to baseline after 6, 13, 26, 39 and 52 weeks of twice daily placebo treatment. **A** PSPRS. **B** Limb-motor domain of PSPRS (fine motor skills, including tremor). **C** Bulbar domain data. **D** Gait midline results. **E** Daily activities. **F** SEADL scale results. Significance, comparing males to females (Wilcoxon test) for a time point (as indicated) is denoted by asterisks, following the same convention as in Figs. 1 and 3, “#” denotes 0.05 < *P* < 0.1. Males (*n* = 55 placebo, *n* = 55 davunetide) and females (*n* = 48 placebo, *n* = 51 davunetide).

Given the observed sex differences in ventricular volume, with considerable volume increases correlating with baseline levels in placebo-treated females alone (Fig. 2), and multiple sex differences (Table S1) also at the correlative level (Fig. 3 and Table S2), we compared clinical disease deterioration in placebo-treated females and males using the primary endpoints, measured over 52 weeks. Figure 4 shows changes in PSPRS at 6, 13, 26, 39 and 52 weeks after study initiation in the placebo groups, thus defining disease progression. A faster disease rate was observed in females than in males, reaching significance at 52 weeks (*P = 0.04; Fig. 4A). As indicated above, PSPRS measures disability across 28 items in six domains, specifically, daily activities (by history), behavior, bulbar activity, ocular motor activity, limb motor activity and gait/midline. As such, we also examined sex-related differences in each of the six domains (Fig. 4B–E and S6). Our results indicated faster deterioration in females in multiple domains, including [1] limb motor activity (fine motor skills and tremor), with *P = 0.02 and 0.01, at weeks 26 and 52, respectively (Fig. 4B), [2] bulbar activity (*P = 0.04 at week 52, Fig. 4C), [3] gait midline (*P = 0.03, at 52 weeks, Fig. 4D), and [4] trending in daily activities (^#^*P* = 0.05, Fig. 4E). Oculomotor and behavior measures showed an almost similar disease progression in males and females (Fig. S5). Furthermore, the most substantial sex differences were observed in SEADL scale deterioration, significantly different as early as at week 13 (*P = 0.01, Fig. 4F), with –*r* = −0.59 and *P* = 1.5e−44 for females and *r* = −0.18, *P* = 0.00014 for males. Together with steeper graph slopes for women (Fig. 4A–E), most pronounced for the SEADL scale endpoint, these findings indicate accelerated disease deterioration in females.

### Davunetide treatment provides women with significant protection against deterioration in the SEADL scale and several PSPRS domains

Given the substantially different disease course seen in males and females (Figs. 2–4), we considered davunetide-mediated protection in males and females separately. In assessing efficacy in males, the davunetide-treated group showed significant increases in PSPRS and decreases in the SEADL scale from week 13 onward, mimicking the more rapid disease progression in females (Fig. S6). This faster deterioration in davunetide-treated males was also apparent, albeit to a lesser degree, in most PSPRS measures (Fig. S6).

**Fig. 5:**
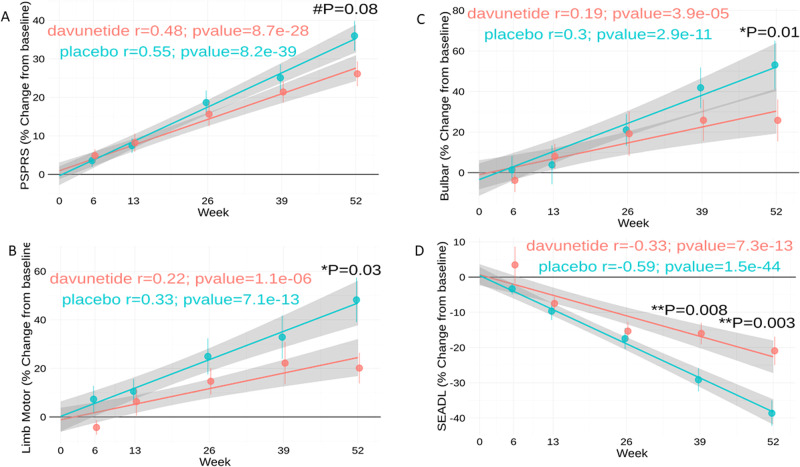
Significant protection against lumbar motor, bulbar and SEADL scale deterioration upon davunetide treatment in women. Linear regression plots (depicting the Pearson correlation coefficient *r*) comparing percent change relative to baseline after 6, 13,26, 39 and 52 weeks in females receiving davunetide or placebo. **A** PSPRS, **B** the limb motor domain of PSPRS, **C** the bulbar domain of PSPRS, and **D** SEADL scale. Significance is denoted by asterisks, following the same convention as in Figs. 1, 3, and 4. Males (*n* = 55 placebo, *n* = 55 davunetide) and females (*n* = 48 placebo, *n* = 51 davunetide).

In contrast, davunetide treatment of females resulted in PSPRS measures that trended toward significance protection at 52 weeks (Fig. 5A, ^#^*P* = 0.08), with an adverse effect on oculomotor activities being seen at week 6, which disappeared thereafter (Fig. S7, *P = 0.01). Notably, oculomotor activities were very similar in males and females (Fig. S5) and a similar adverse trending effect was seen in both (Fig. S6, ^#^*P* = 0.09). Unlike what was seen with males, females did not present adverse effects in any other domains (Figs. 5A–D and S7). Importantly, while significant deterioration was noted in placebo-treated female mental behavior, as reflected by disorientation, bradyphrenia, including slowness of thought processes and inattentiveness, emotional incontinence and grasping behavior affecting activities of daily living, upon davunetide treatment, disease progression was insignificant in terms of effects on mental behavior (davunetide, *r* = 0.29, placebo, *r* = 0.37, *P* < 0.0001; Fig. S7). Furthermore, a significant davunetide-mediated protective effect on the limb-motor domain (Fig. 5B, week 52, *P = 0.03) and the bulbar domain of the PSPRS (Fig. 5C, week 52, *P = 0.01) against dysarthria, namely, difficulty speaking caused by brain damage, which results in an inability to control the muscles used in speech, and dysphagia, namely, difficulty swallowing. Lastly, the efficacy of davunetide in females was highly significant in the SEADL scale, showing a protective effect starting at week 39, and continuing to week 52 (**P = 0.008 and **P = 0.003, respectively; Fig. 5D).

In summary, we discovered female-specific davunetide target engagement and baseline/age-correlated protection against increases in ventricular volume, coupled with significant effectiveness on limb motor activities (fine motor function, including tremor), functional bulbar activities (i.e., speaking and swallowing) in the PSPRS and apparently no significant deterioration of mental activities, correlated with activities of daily living (SEADL scale), after twice daily davunetide treatment for 52 weeks.

## Discussion

The surprising female-specific neurodegeneration observed as increases in ventricular volume (depending on the baseline starting point) described in the current study is in agreement with recent data showing that the rate of Tau aggregate accumulation is higher in females than in males [48]. Thus, differential protection brought about by different lengths of treatment or dosages assigned need to be considered when treating with microtubule-interacting drugs. Until now, there was no mention of sex-specific effects with regard to davunetide treatment in PSP [28, 47, 49]. However, as stated in the Introduction, increases in the level of filamin A in the PSP brain are associated with Tau pathology [35] through actin interactions, which may be modified by davunetide treatment [6]. Moreover, a filamin A fragment localizes to the nucleus, where it regulates androgen receptor and co-activator functions, implying sexual dichotomy [36]. The filamin A previous findings, coupled with estrogen-mediated regulation of alternative splicing [34], misbalances in 4R and 3R Tau levels leading to differences in axonal transport [50], and the higher susceptibility of microtubules in the female brain to neurodegeneration [14] may explain the vulnerability of the female PSP brain and the significant differences seen, relative to males. Here, we have revealed a sex-dependent differential susceptibility of ventricular volume measure, indicative of accelerated neurodegeneration in female PSP patients, as compared to males. We further discovered differential disease development in men and women, with the disease progressing faster in women, specifically in what seems to be fine motor deterioration, activities of daily living and mental functions. Additionally, the PSP female patient brain reacted differently to davunetide treatment than did the PSP male brain, as seen by the age- and baseline volume-dependent manners of response, which require neuroprotection, with davunetide treatment protecting microtubule-actin interactions [6], brain structure [51], and axonal transport [27], regulated by ADNP in a sex-dependent manner [2].

Falls within 1 year of disease onset, apathy, and executive dysfunction are more frequent in female PSP patients. Similarly, the time needed for unintelligible speech, severe dysphagia and cognitive impairment to appear is shorter in females [37]. These previous findings agree with the current study’s SEADL scale correlation with brain ventricular volume changes in females but not in males, male-female differences in SEADL scale progression, and the davunetide-mediated bulbar and RBAN language protection only seen in females. Along the same lines, another recent study showed that basal metabolism was lower for the PSP group, as opposed to the women-only group. This study also showed significant correlation between skeletal mass index and age, basal metabolism, the extracellular water/total body water ratio, and body fat percentage only in the female PSP group [52]. In this respect, ADNP/davunetide (NAP) treatment regulates mouse developmental milestones, gait, spleen gene expression and microbiome composition in a sex-dependent manner [5].

In considering male subjects, mirror images of the effects of davunetide treatment in females were most apparent in SEADL scale measurements (Figs. 4 and S6). Male-specific effects may be due to higher male brain ADNP expression, as compared to females [13, 39], with davunetide complementing deficient ADNP, such that over-expression of ADNP may result in unwanted overdose side effects [7]. In this respect, brain ADNP expression is regulated by the estrus cycle [39] and a recent study suggested that early onset of menopause and late initiation of hormone therapy following menopause onset may be important sex-specific risk factors that underlie sex differences in Tau deposition. Accordingly, women exhibiting premature menopause (i.e., before the age of 40 years) showed higher Tau deposition levels than women exhibiting early menopause at 40–45 years of age. Both groups showed higher Tau deposition levels, as compared to women who experienced menopause at >45 years of age [41]. With ADNP regulating sex steroids [10], and with davunetide able to compensate for ADNP deficiencies [11], these findings emphasize the requirement for dose selection appropriate for the study population.

Our findings are also in line with sex differences associated with the blood-brain barrier (BBB). Decreased BBB efflux function of the P-glycoprotein transport system was observed in PSP [53], whereas age- and sex-dependent effects were described in healthy individuals, with this effect significantly decreasing in men as they aged [54]. Thus, the davunetide dosage used with men in the present study was excessive.

Regardless, the observed correlation of behavioral outcomes with ventricular volume protection is of high significance. Indeed, a study published in 2008 identified ventricular enlargement as a possible measure of AD progression, as validated using the AD neuroimaging initiative (ADNI) database. The major conclusion of that study was that the number of subjects demonstrating a 20% change in ventricular enlargement was substantially lower than that required to demonstrate a 20% change in cognitive scores. Ventricular enlargement thus represents a feasible short-term marker of disease progression in subjects with MCI and subjects with AD for multi-center studies [55]. A more recent review highlighted progressive dose-dependent ventricular enlargement in the brains of patients with early AD subjected to treatment with the FDA-approved drug aducanumab. Aducanumab joins a growing list of anti-amyloid-β therapies for which evidence supports their causing accelerated neurodegeneration. Still, extended follow-up studies are required to determine whether or not aducanumab causes progressive brain damage [56]. Our current findings imply that davunetide can protect against this unwanted potential side effect of amyloid-targeting drugs, as well as protect against disease-related ventricular volume increases, presenting a first-of-its-kind drug with human efficacy in women suffering from PSP, and possibly beyond.

An obvious limitation of the current study is whether there was clinically important improvement upon davunetide treatment. An earlier study that considered all of the davunetide-related results together [49] (despite the statistically significant sex differences shown here) suggested that the minimal clinically important worsening in PSPRS was 5.7 points, corresponding to the mean decline over six months in the trial (i.e. about a 5% change over 6 months). Here, after 52 weeks, davunetide afforded a significant mean of about 20% protection in limb motor and bulbar deterioration in the female population (as compared to no significant protection after six months without such treatment), suggestive of a potential meaningful clinical impact. Additionally, the davunetide-mediated inhibition of SEADL scale deterioration was about 10% (already significant at 39 weeks of treatment), suggesting meaningful clinical efficacy, cautiously coupled to neuroprotection and/or target engagement.

From a translational medicine point of view, our findings are also in agreement with results obtained with mouse models of the autistic/intellectual disability known as ADNP syndrome in which patients suffer from speech delays and language impairment because of *ADNP* mutations. In that case, davunetide (NAP) treatment protected against ADNP deficiency-related vocalization/syntax effects in mice [5, 11]. Mechanistically, again as indicated in the Introduction, NAP was also shown to interact with the armadillo motif of beta-catenin [7, 8], further regulating forkhead box P2 (FOXP2), a highly conserved transcription factor essential for language and speech development [57]. Our previous work has shown ADNP/NAP-mediated regulation of FOXP2 in the tongue [58] and NAP-mediated regulation of FOXP2 in brain [59]. Interestingly, a recent study indicated sexual dimorphism in the relationship between FOXP2 and body-mass index with cognitive deficits in schizophrenia [60]. Importantly, our present findings are in accord with the results of previous clinical studies on the usefulness of davunetide in schizophrenia, where a significant effect on the co-primary endpoint, functional activities of daily living [16], coupled to neuroprotection [61], was seen. Furthermore, attesting to davunetide-mediated brain protective effects, a mutation in the davunetide sequence within ADNP (NAPVSIPQQ to NAPVSIPQE) causes a mild neurodevelopmental syndrome affecting brain function [8].

An obvious strength of the study is the separation of results obtained from men and women in the clinical study, which agrees with accumulating literature emphasizing brain disease-associated gender differences [62–65]. Specifically, the tendency of higher Tau aggregate accumulation in women has also been reported for other neurodegenerative diseases, such as AD [66], which may result from sex differences in microglia networks and age-dependent loss (for example, in AD [67] and PSP [68]).The surprising sex differences in PSP led to the discovery of the significant clinical efficacy of davunetide in women, correcting the initial assumption of sex similarity in PSP disease progression [28].

In conclusion, evaluating the results of the present study in more general terms accentuates the requirements for detailed analysis, especially in complex diseases and syndromes of the central nervous system, which potentially present several converging pathways. We highly recommend that all clinical trials, retrospectively and prospectively, separately analyze males and females, paying close attention to disease progression, dose, age, and baseline characteristics, toward precision medicine. Accordingly, the current study showed, for the first time, davunetide-target engagement and possible protection against neurodegeneration, coupled with statistically significant clinical efficacy, in female PSP patients. We thus propose that davunetide should be developed for use with women suffering from PSP.

### Supplementary information

Supplemental Material

Table S1

Table S2
