# Supplementary material for: Unexpected gender differences in progressive supranuclear palsy reveal efficacy for davunetide in women
Source: Transl Psychiatry. 2023 Oct 16;13:319. doi: 10.1038/s41398-023-02618-9 (PMC10579238; doi:10.1038/s41398-023-02618-9)
Supplement: Supplementary file 1 — Supplemental Material [file 41398_2023_2618_MOESM1_ESM.pdf]

Unexpected Gender Differences in Progressive Supranuclear Palsy Reveal Efficacy for Davunetide in Women

Illana Gozes[1,*,**], Guy Shapira[2,*], Alexandra Lobyntseva[1,*], Noam Shomron[2]

[1]Elton Laboratory for Molecular Neuroendocrinology, Department of Human Molecular Genetics and Biochemistry, Faculty of Medicine, Adams Super Center for Brain Studies and Sagol School of Neuroscience, Tel Aviv University, Tel Aviv 69978, Israel
[2]Department of Cell and Developmental Biology, Faculty of Medicine, Sagol School of Neuroscience, Edmond J Safra Center for Bioinformatics, Tel Aviv University, Tel Aviv 69978, Israel

*Contributed equally

**Correspondence to:
Professor Illana Gozes, Elton Laboratory for Molecular Neuroendocrinology, Department of Human Molecular Genetics and Biochemistry, Faculty of Medicine, Adams Super Center for Brain Studies and Sagol School of Neuroscience, Tel Aviv University, Tel Aviv 69978, Israel. E-mail: igozes@tauex.tau.ac.il

Supplementary Materials:

Figure S1:
Contributing clinicians and scientists to study design and execution:
Adam L Boxer[1], Anthony E Lang[2], Murray Grossman[3], David S Knopman[4], Bruce L Miller[5], Lon S Schneider[6], Rachelle S Doody[7], Andrew Lees[8], Lawrence I Golbe[9], David R Williams[10], Jean-Cristophe Corvol[11], Albert Ludolph[12], David Burn[13], Stefan Lorenzl[14], Irene Litvan[15], Erik D Roberson[16], Günter U Höglinger[17], Mary Koestler[5], Clifford R Jack Jr[18], Viviana Van Deerlin[19], Christopher Randolph[20], Iryna V Lobach[5], Hilary W Heuer[5], Illana Gozes (corresponding author of the current post hoc analysis)[21], Lesley Parker[22], Steve Whitaker[23], Joe Hirman[24], Alistair J Stewart[25], Michael Gold[26], Bruce H Morimoto[27], AND AL-108-231 Investigators[28]

Affiliations in when Boxer et al. (2014)[1] was published:
[1]Memory and Aging Center, Department of Neurology, University of California, San Francisco, CA, USA Corresponding author, electronic address: aboxer@memory.ucsf.edu.
[2]Department of Neurology, University of Toronto, Toronto, ON, Canada
[3]Department of Neurology, University of Pennsylvania, Philadelphia, PA, USA
[4]Department of Neurology, Mayo Clinic, Rochester, MN, USA
[5]Memory and Aging Center, Department of Neurology, University of California, San Francisco, CA, USA
[6]Department of Psychiatry and the Behavioural Sciences and Department of Neurology, Keck School of Medicine of the University of Southern California, Los Angeles, CA, USA
[7]Department of Neurology, Baylor College of Medicine, Houston, TX, USA
[8]Institute of Neurology, University College London, UK
[9]Department of Neurology, Rutgers Robert Wood Johnson Medical School, New Brunswick, NJ, USA
[10]Faculty of Medicine, Monash University, Melbourne, Victoria, Australia
[11]Assistance Publique-Hôpitaux de Paris, INSERM, CIC1422 and UMRS1027, Sorbonne Universités, Université Pierre et Marie Curie, Paris, France; Department of Neurology, Pitié-Salpêtrière Hospital, Paris, France
[12]Department of Neurology, University Hospital, Ulm, Germany
[13]Institute for Ageing and Health, Newcastle University, Newcastle, UK
[14]Interdisciplinary Center for Palliative Medicine, Munich University Hospital-Klinikum Grosshadern, Munich, Germany
[15]Department of Neurology, University of California, San Diego, CA, USA
[16]Department of Neurology, University of Alabama, Birmingham, AL, USA
[17]Department of Translational Neurodegeneration, Technical University Munich, Munich, Germany.
[18]Department of Radiology, Mayo Clinic, Rochester, MN, USA
[19]Department of Neurology and Pathology and Laboratory Medicine, Perelman School of Medicine, University of Pennsylvania, Philadelphia, PA, USA
[20]Department of Neurology, Loyola University School of Medicine, Chicago, IL, USA
[21]Department of Human Molecular Genetics and Biochemistry, Sackler Faculty of Medicine and Sagol School of Neuroscience, Adams Super Center for Brain Studies, Tel Aviv University, Tel Aviv, Israel
[22]Tekmira Pharmaceuticals, Burnaby, BC, Canada
[23]Omeros, Seattle, WA, USA
[24]Pacific Northwest Statistical Consulting, Woodinville, WA, USA
[25]Paladin Laboratories, St Laurent, QC, Canada
[26]UCB BioSciences, Research Triangle Park, NC, USA
[27]Celerion, Lincoln, NE, USA
[28]AL-108-231 Investigators: David Williams, Anne Louise Lafontaine, Connie Marras, Mandar Jog, Michael Panisset, Jean-Christophe Corvol, Jean-Philippe Azulay, Philippe Couratier, Brit Mollenhauer, Stefan Lorenzl, Albert Ludolph, Reiner Benecke, Günter Höglinger, Axel Lipp, Heinz Reichmann, Dirk Woitalla, Dennis Chan, Adam Zermansky, David Burn, Adam Boxer, Erik Roberson, Lawrence Honig, Edward Zamrini, Rajesh Pahwa, Yvette Bordelon, Erika Driver-Dunkley, Stephanie Lessig, Mark Lew, Kyle Womack, Brad Boeve, Joseph Ferrara, Argye Hillis, Daniel Kaufer, Rajeev Kumar, Tao Xie, Steven Gunzler, Theresa Zesiewicz,

Praveen Dayalu, Lawrence Golbe, Murray Grossman, Joseph Jancovic, Scott McGinnis, Anthony Santiago, Paul Tuite, Stuart Isaacson, Julie Leegwater-Kim, Irene Litvan

Figure S1: Study design diagram

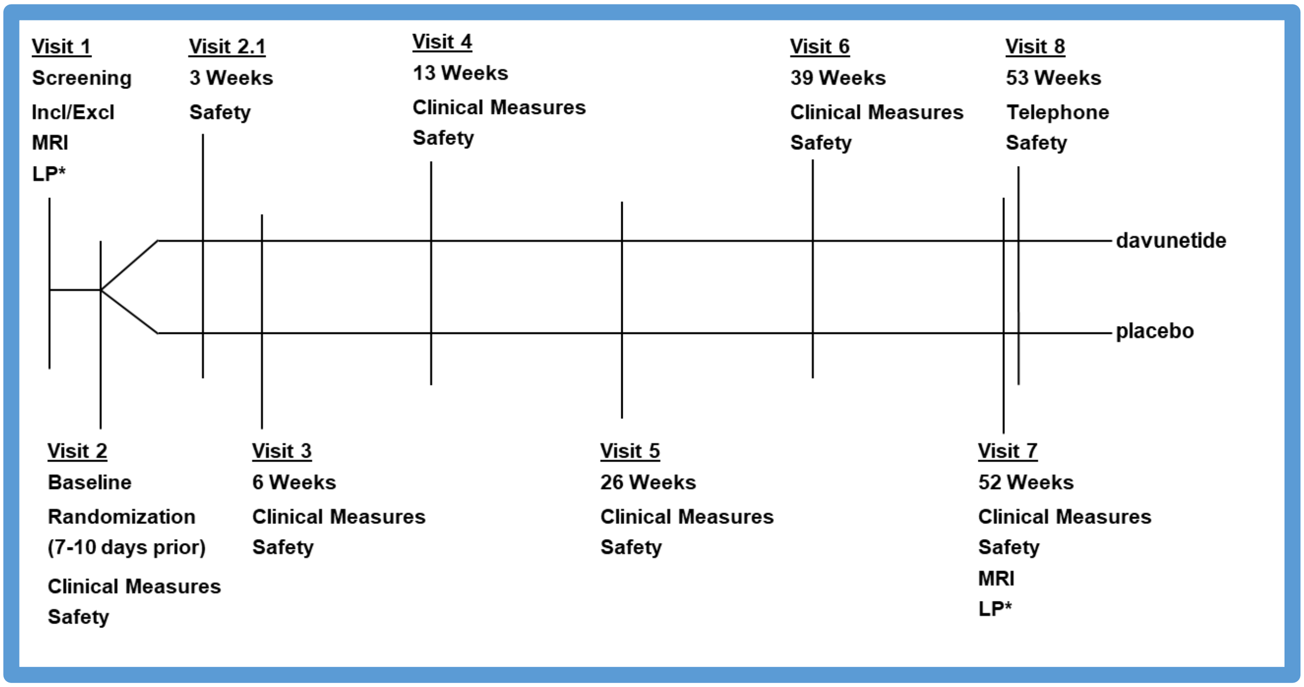

*LP = Lumbar puncture

List of Excel Tables:

Excel Table S1: All group-wise comparisons of assessment measures, results from a Wilcoxon test of change from baseline at week 52. Each row lists the variable tested, groups of the comparison, their respective sample size (n1, n2), the test statistic and P value.

Excel Table S2: Full table of correlations between age and the fractional difference in nine different measurements. Each row represents a Pearson correlation test between two variables in a single group, followed by the correlation coefficient, statistic, P value and confidence interval (cor=correlation, var=variable).

Figure S2:

Trending significant davunetide-mediated protection against ventricular volume increases is apparent only in female PSP patients

Ventricular volume changes across the entire study population showing davunetide-mediated protection only in females (n=48, placebo; n=51, davunetide; #P=0.05; Wilcoxon test, box plot). No protection was observed in males (n=55, placebo; n=55, davunetide, P>0.4, Wilcoxon test, box plot).

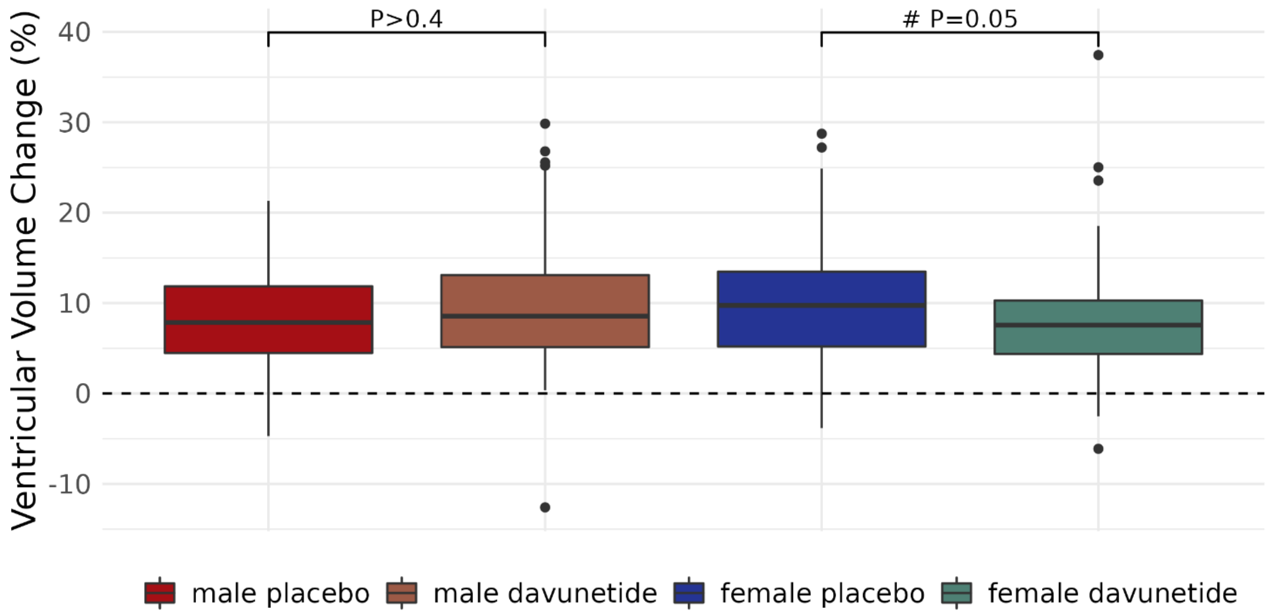

Figure S3:

Individual differences in davunetide-mediated ventricular volume protection in males and females suffering from PSP.
Ventricular volumes are shown in mm^3 for all tested individuals.

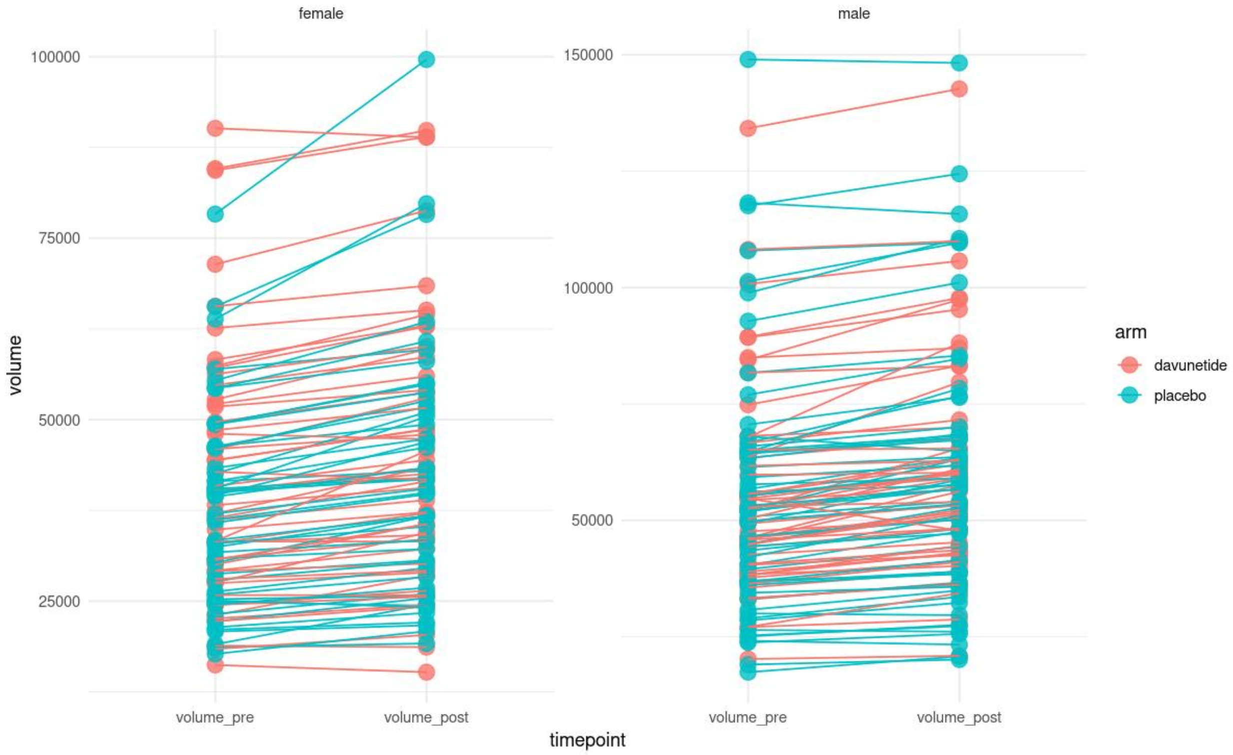

Figure S4:

Potential changes in cerebrospinal fluid Tau levels.
The available lumbar puncture (LP) data for the original cohort was too small to reveal treatment-significant changes, although a trend was seen. Graphs (Prism) indicate differences, Student’s T test.

Total Tau difference between placebo and davunetide in females

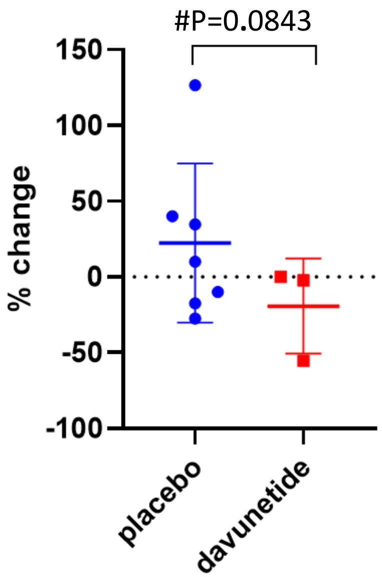

Phosphorylated Tau in females

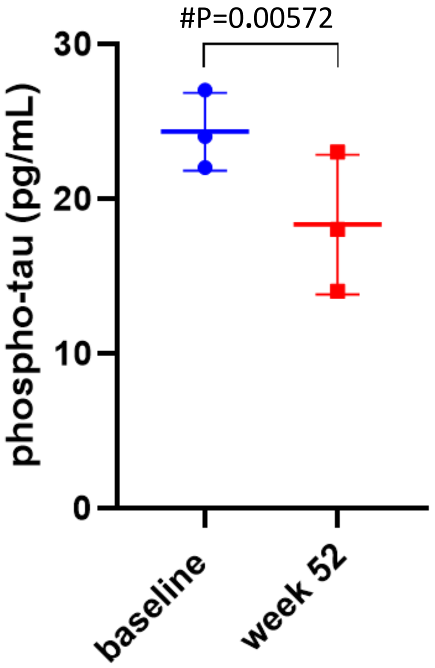

Figure S5:

PSP develops more rapidly in females, addressing all PSPRS domains.
Only the placebo groups were assessed. Significant changes are shown in Fig. 4 (females, red line).

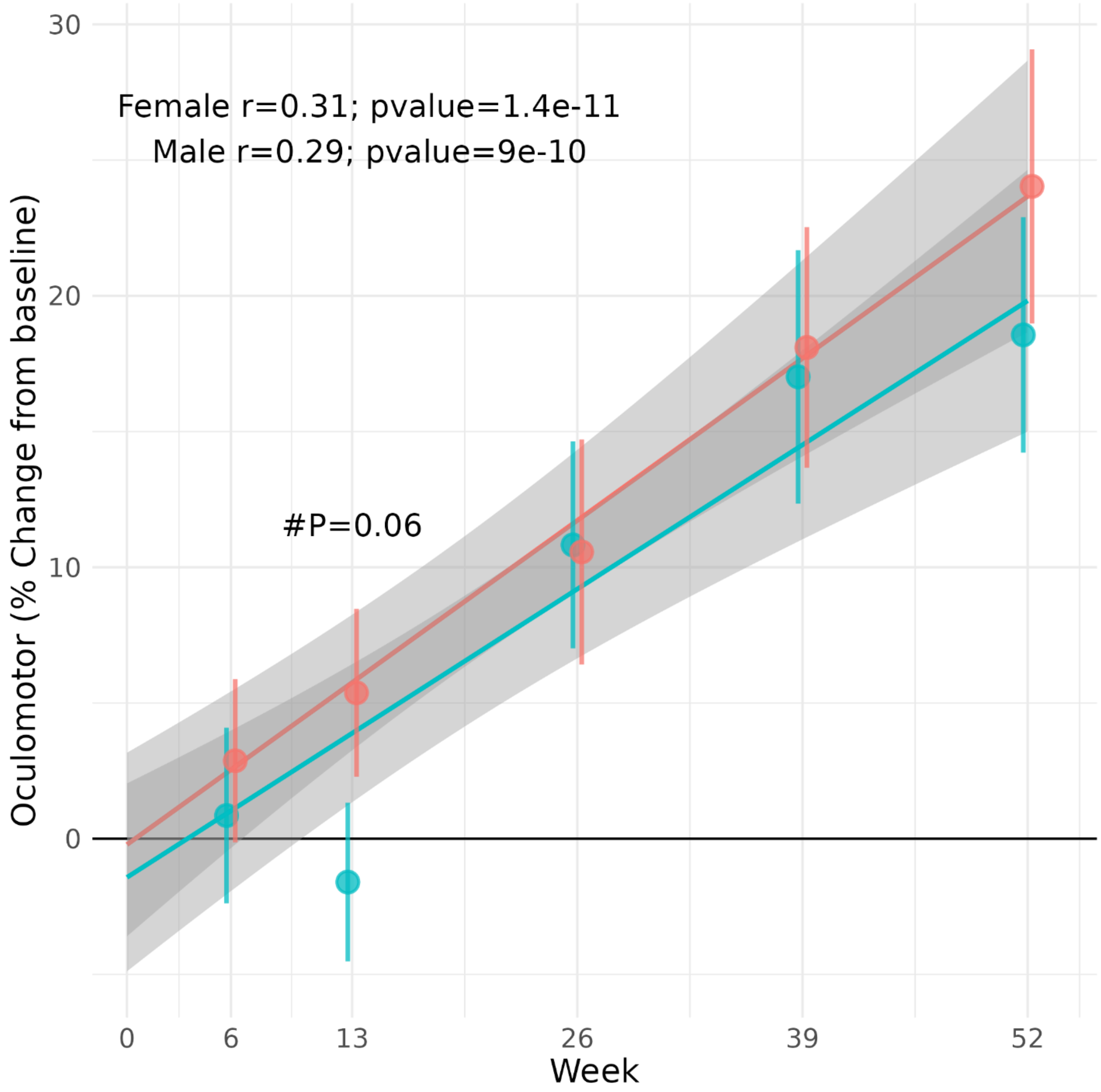

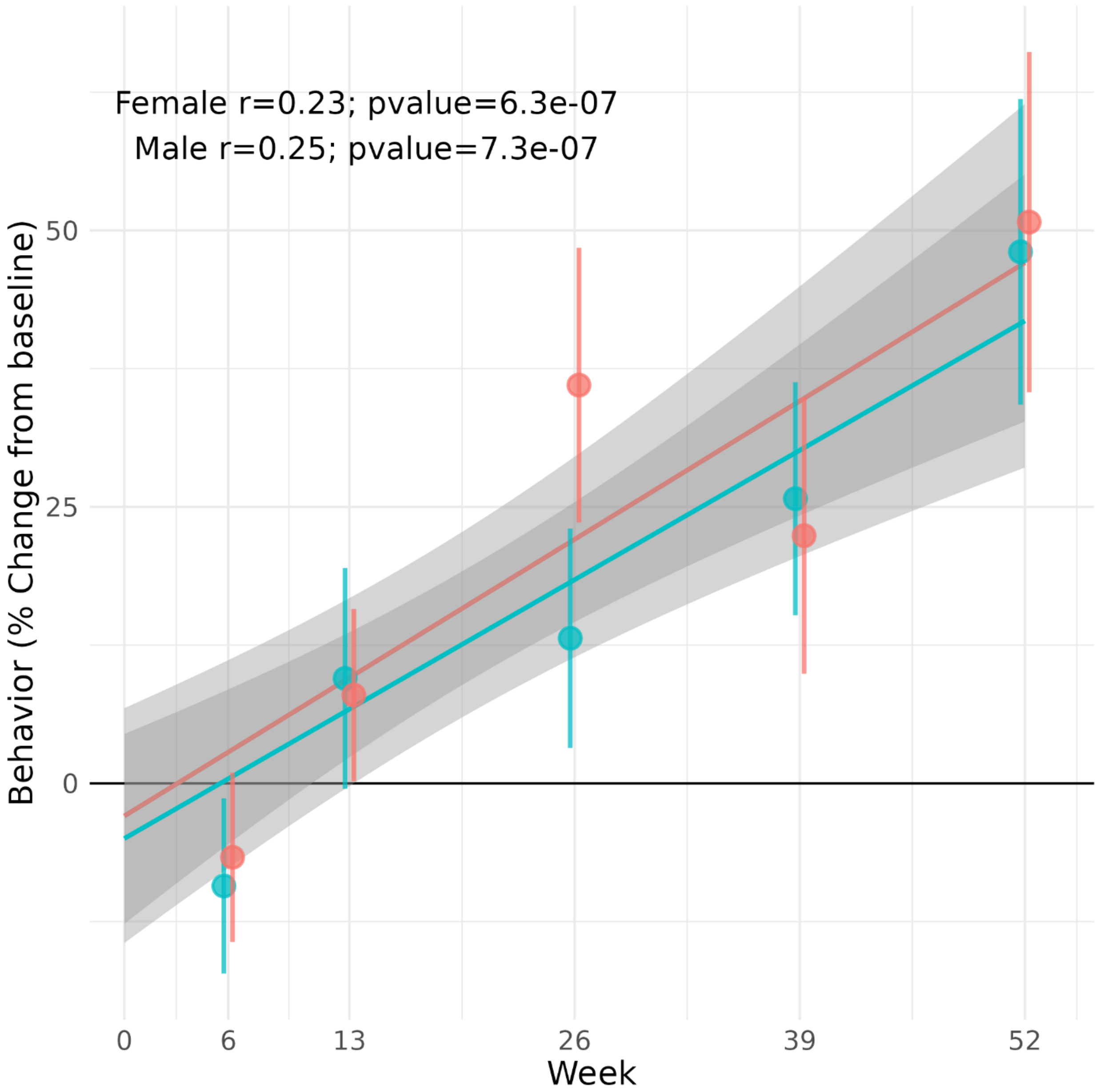
Female r=0.23; pvalue=6.3e-07
Male r=0.25; pvalue=7.3e-07
Behavior (% Change from baseline)
50
25
0
0
6
13
26
39
52
Week

Figure S6:

Davunetide treatment does not protect men against PSPRS and SEADL deterioration. The figure is like Fig. 5, with only men being shown, instead of women. Top: PSPRS, including all tested domains. Bottom: SEADL (davunetide, red line). Wilcoxon statistics are shown, per time point.

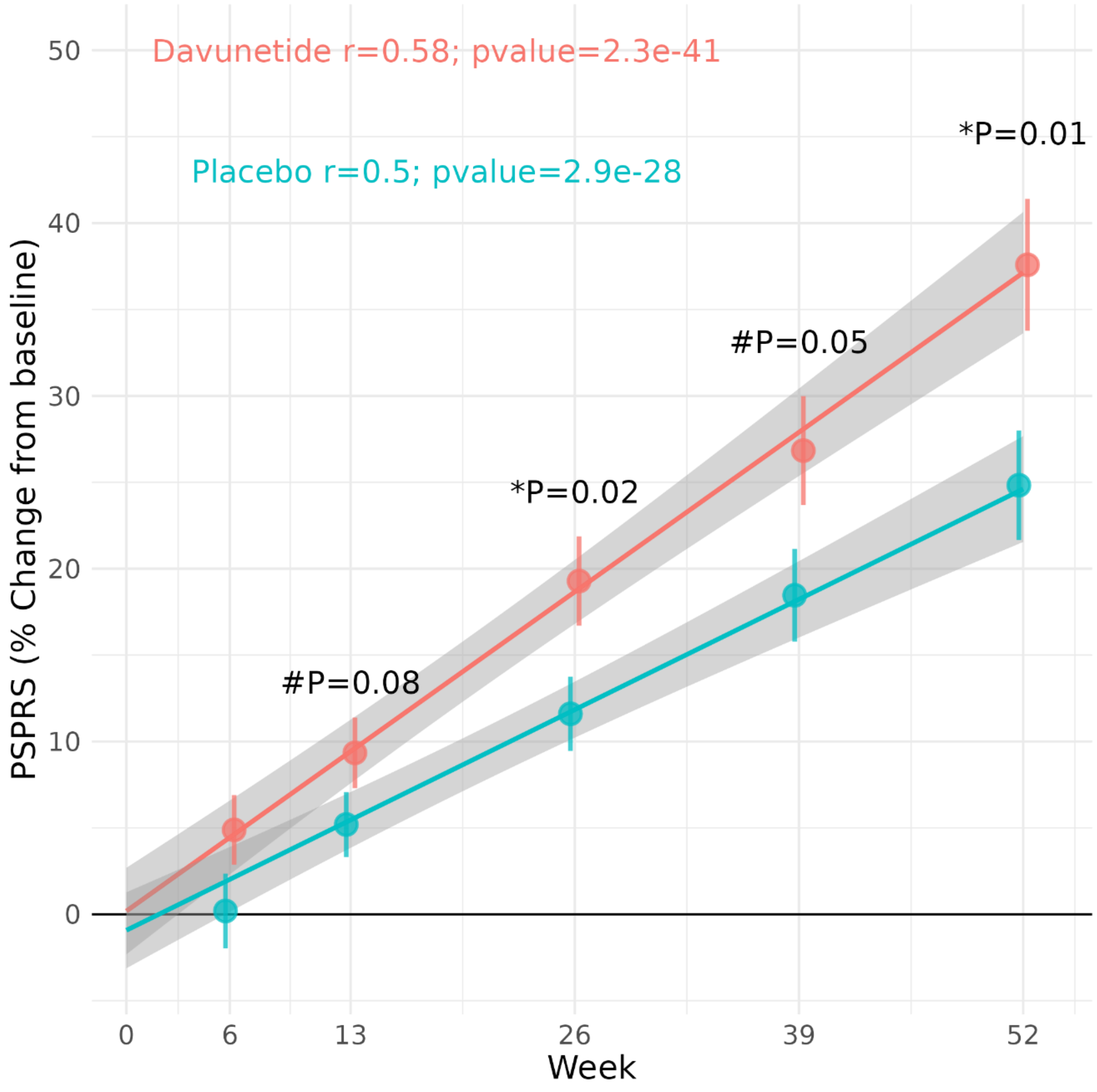

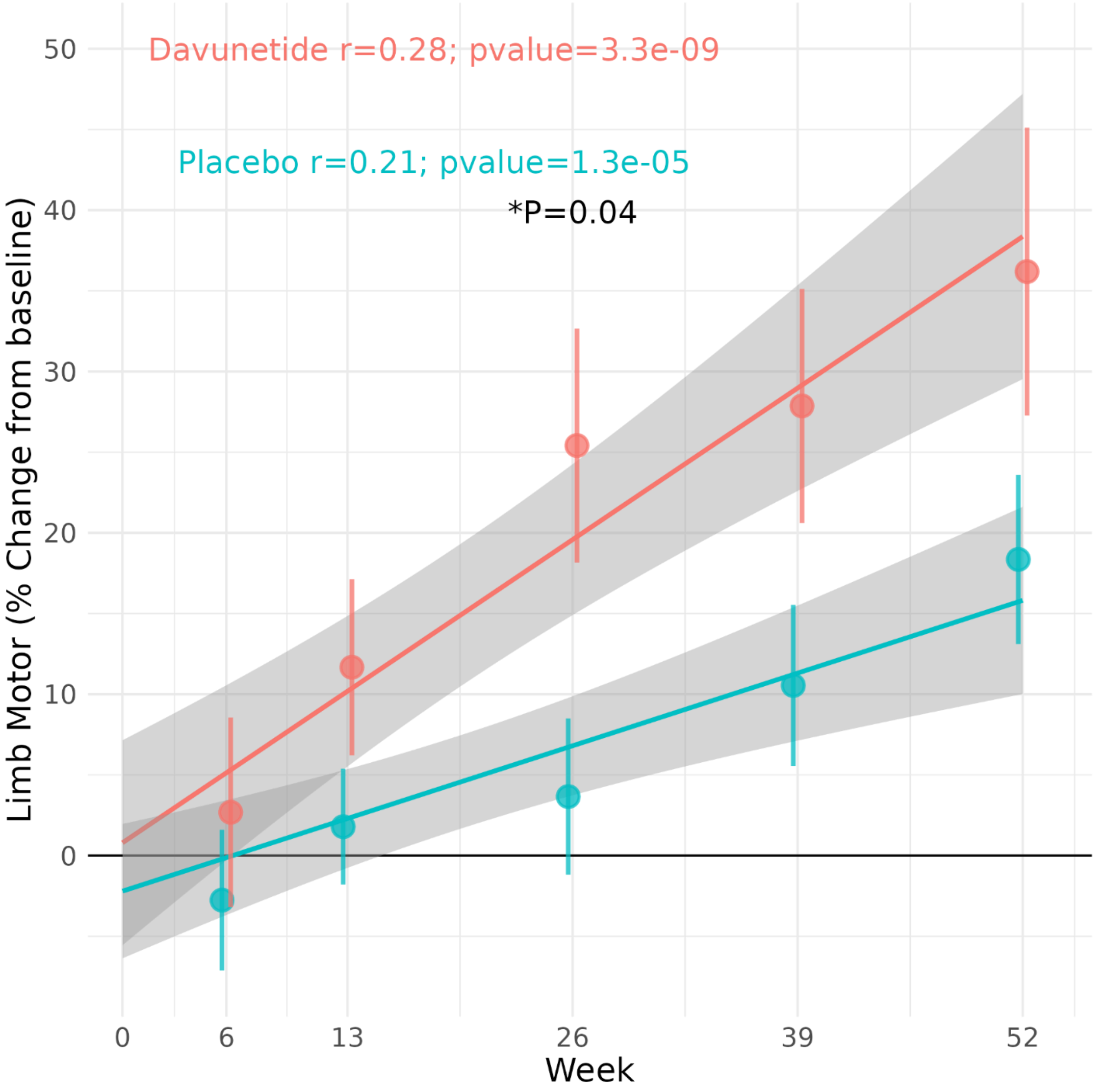
Davunetide r=0.28; pvalue=3.3e-09
Placebo r=0.21; pvalue=1.3e-05
*P=0.04
Limb Motor (% Change from baseline)
50
40
30
20
10
0
0
6
13
26
39
52
Week

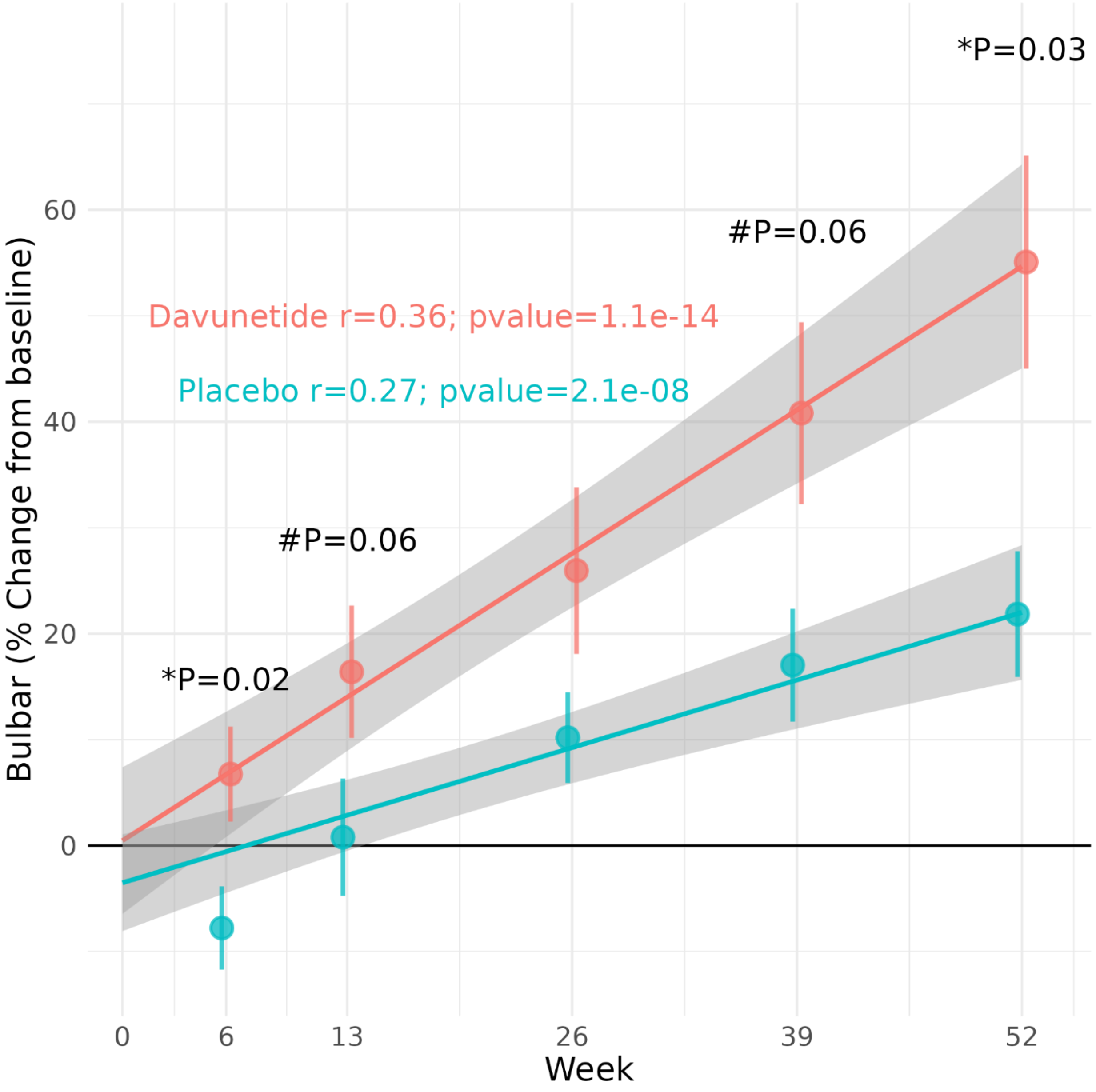
*P=0.03
#P=0.06
Davunetide r=0.36; pvalue=1.1e-14
Placebo r=0.27; pvalue=2.1e-08
#P=0.06
*P=0.02
Bulbar (% Change from baseline)
60
40
20
0
0
6
13
26
39
52
Week

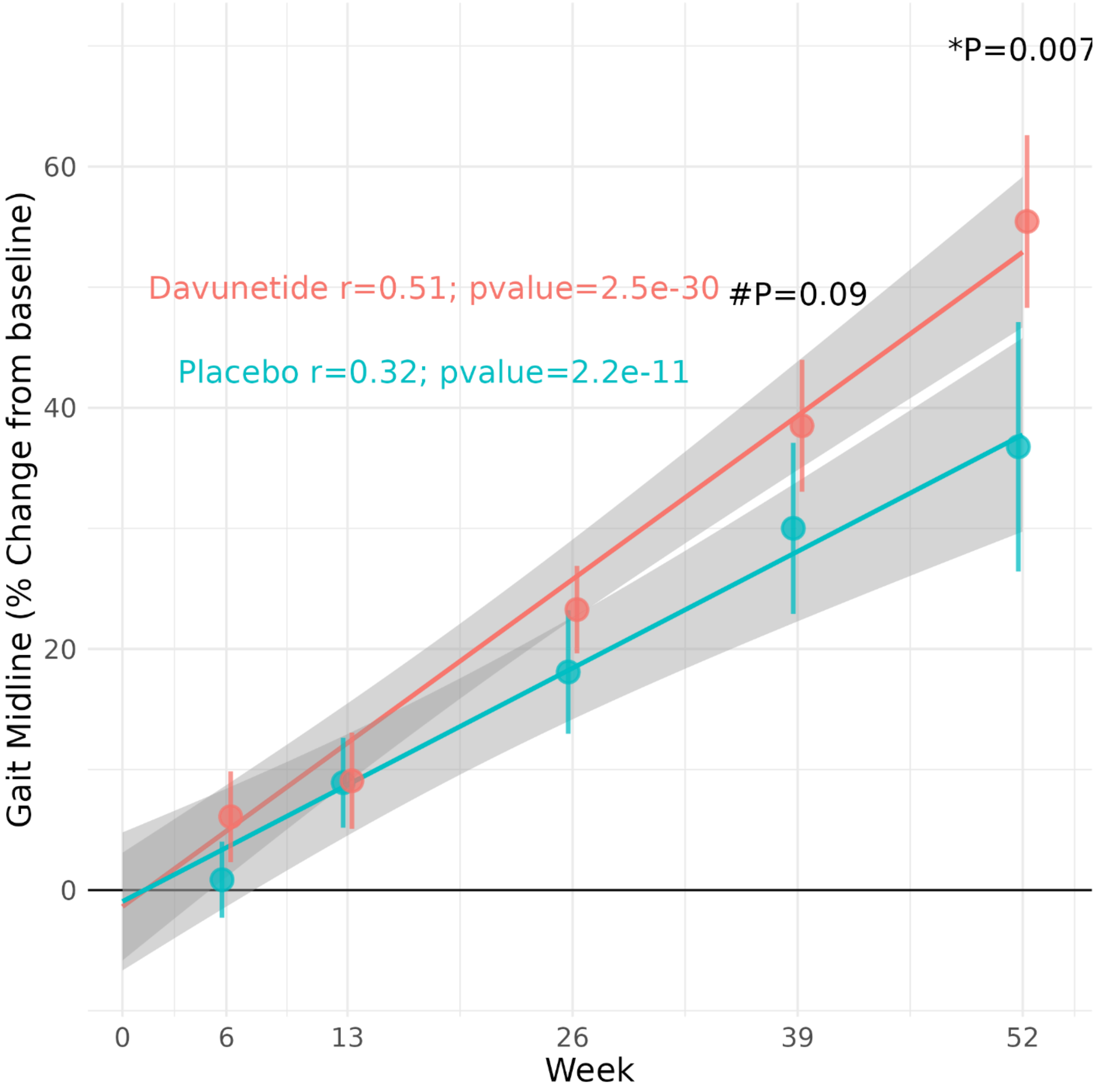
*P=0.007
Davunetide r=0.51; pvalue=2.5e-30
#P=0.09
Placebo r=0.32; pvalue=2.2e-11
Gait Midline (% Change from baseline)
60
40
20
0
0
6
13
26
39
52
Week

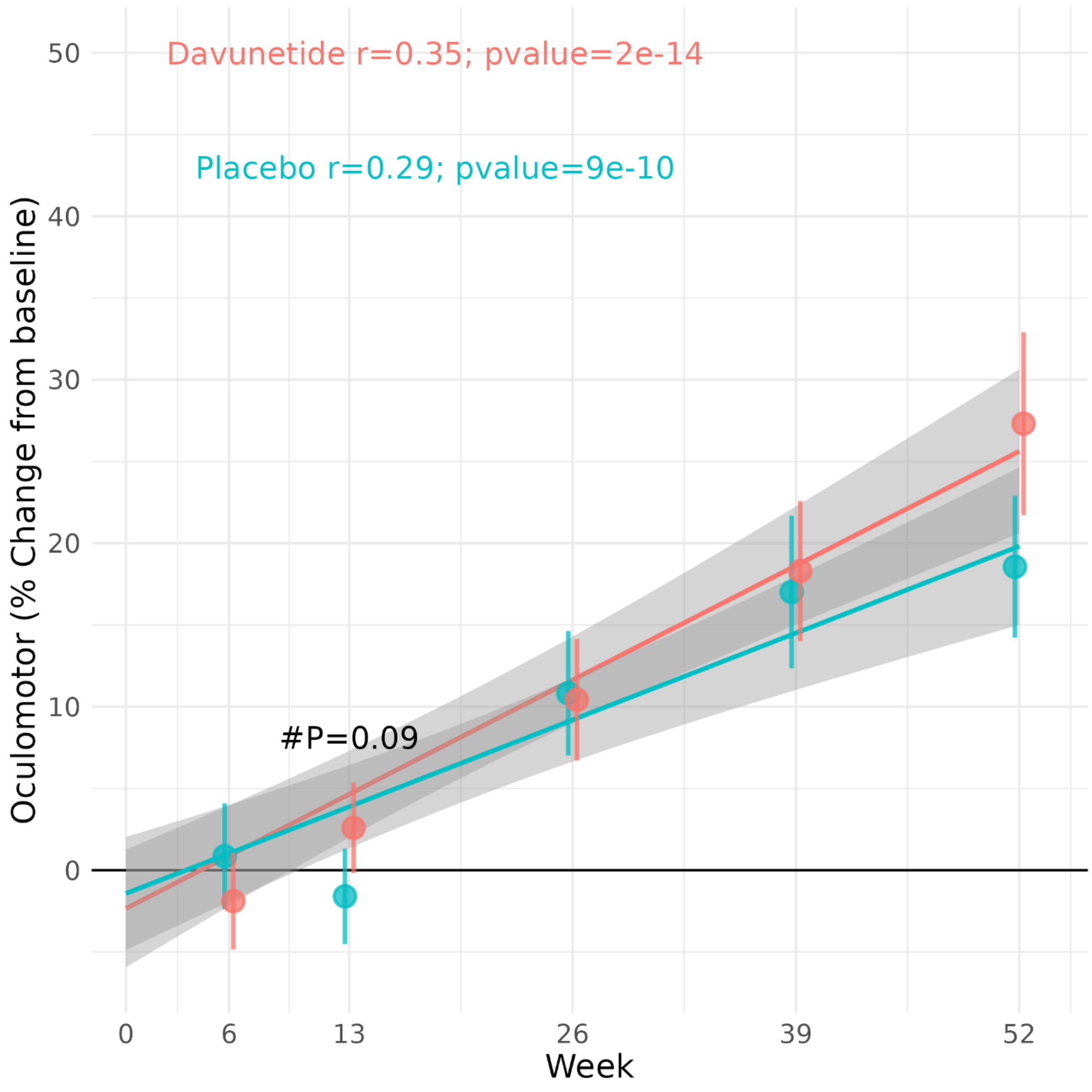
Davunetide r=0.35; pvalue=2e-14
Placebo r=0.29; pvalue=9e-10
#P=0.09
Oculomotor (% Change from baseline)
Week
0
10
20
30
40
50
0
6
13
26
39
52

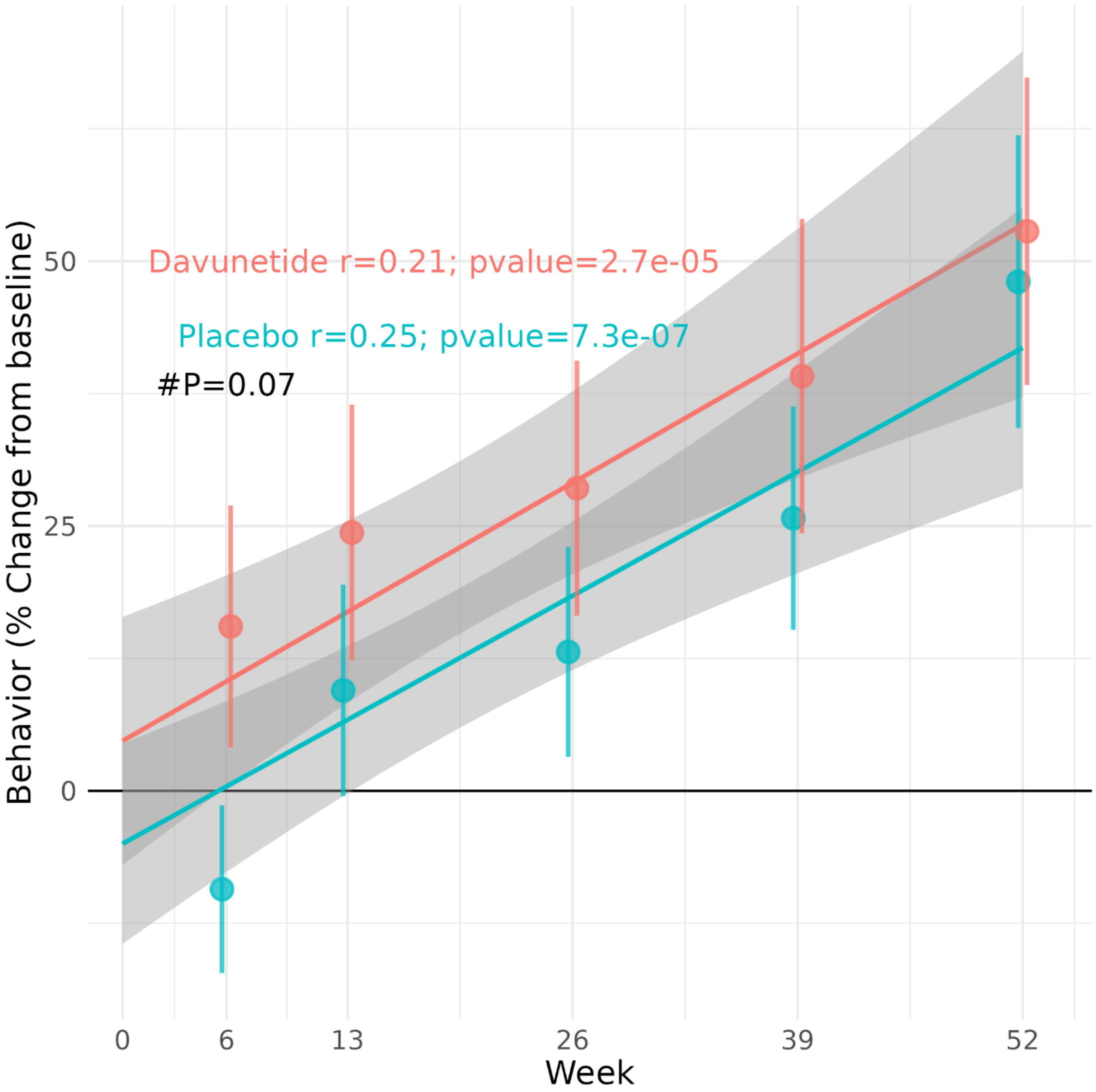
Davunetide r=0.21; pvalue=2.7e-05
Placebo r=0.25; pvalue=7.3e-07
#P=0.07
Behavior (% Change from baseline)
50
25
0
0
6
13
26
39
52
Week

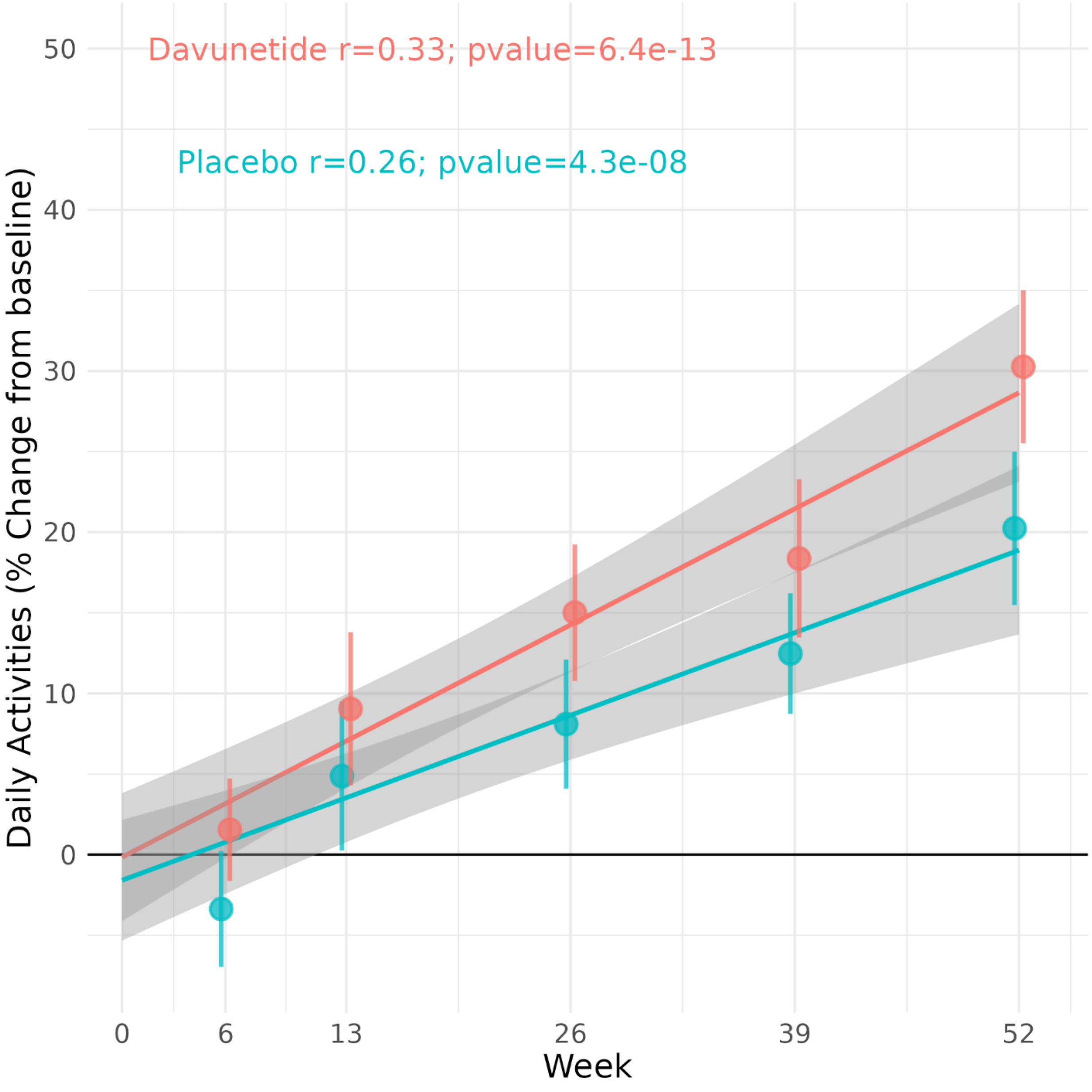
Davunetide r=0.33; pvalue=6.4e-13
Placebo r=0.26; pvalue=4.3e-08
Daily Activities (% Change from baseline)
50
40
30
20
10
0
0
6
13
26
39
52
Week

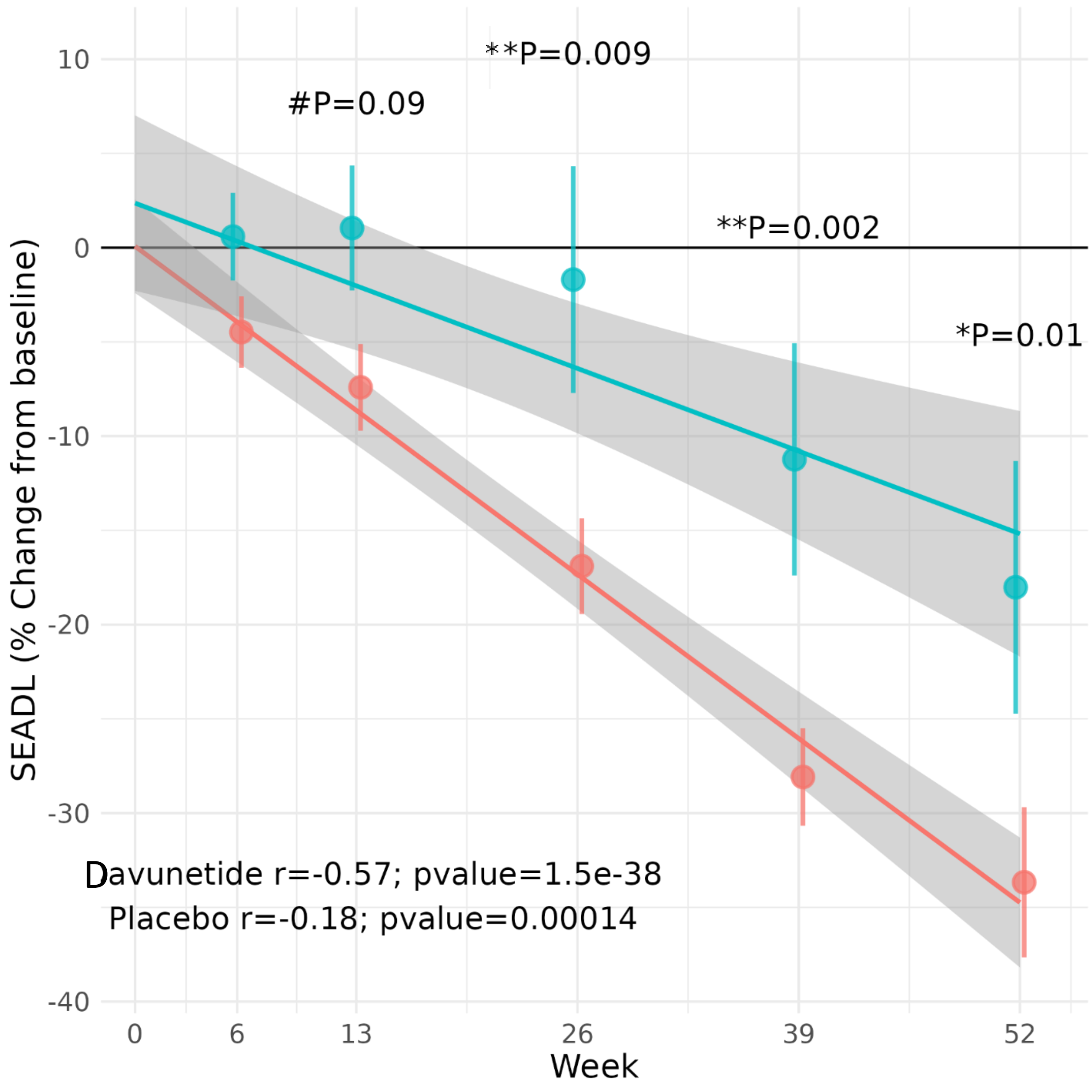

**P=0.009
#P=0.09
**P=0.002
*P=0.01
SEADL (% Change from baseline)
10
0
-10
-20
-30
-40
0
6
13
26
39
52
Week
Davunetide r=-0.57; pvalue=1.5e-38
Placebo r=-0.18; pvalue=0.00014

Figure S7:

Domains of PSPRS protection and trends in protection in females upon davunetide treatment.

Four domains of the PSPRS are shown here, namely, gait/midline, ocular motor, limb motor, daily activities (by history), and behavior (limb motor and bulbar measures are shown on Fig. 5, main text).

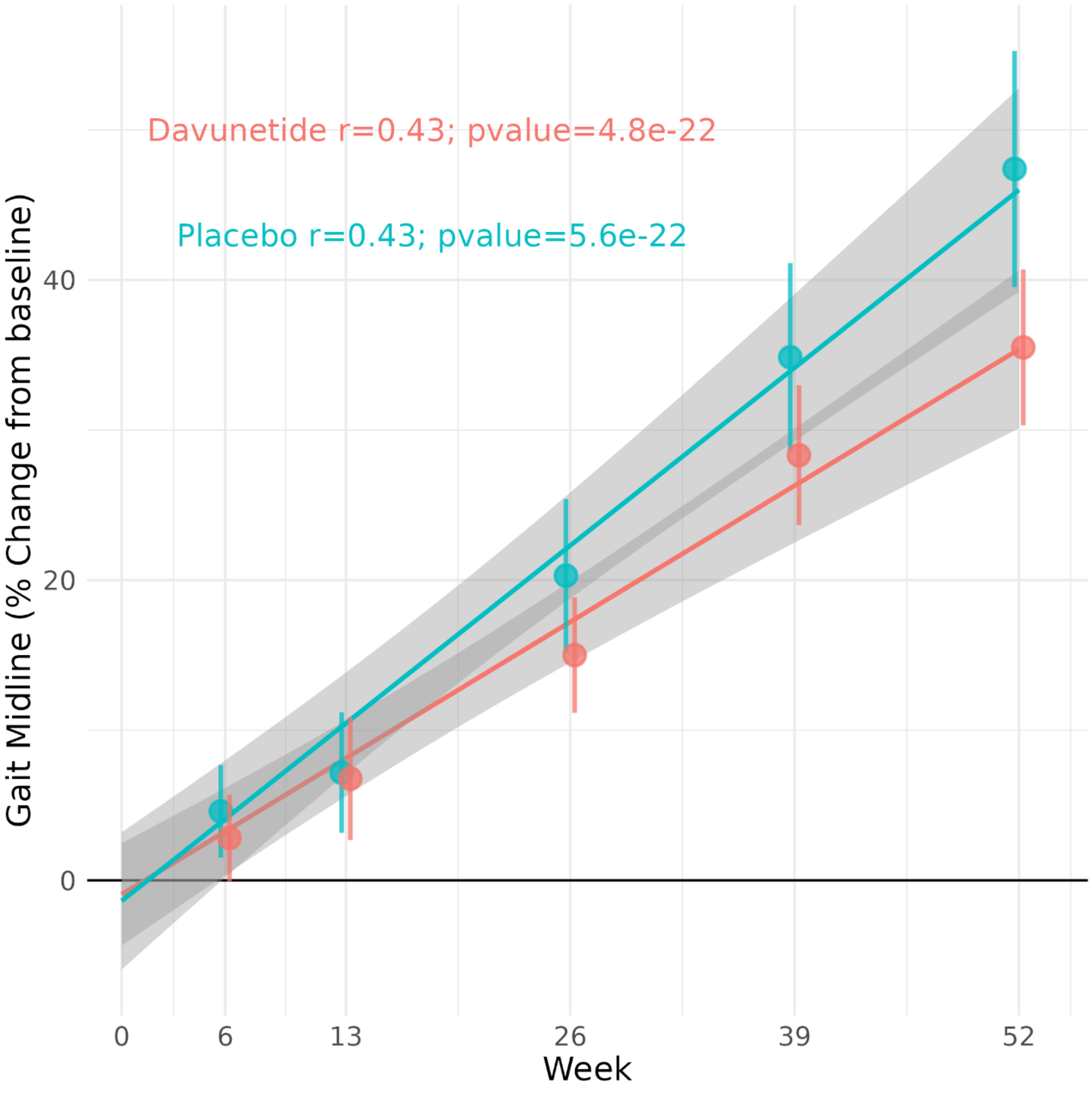

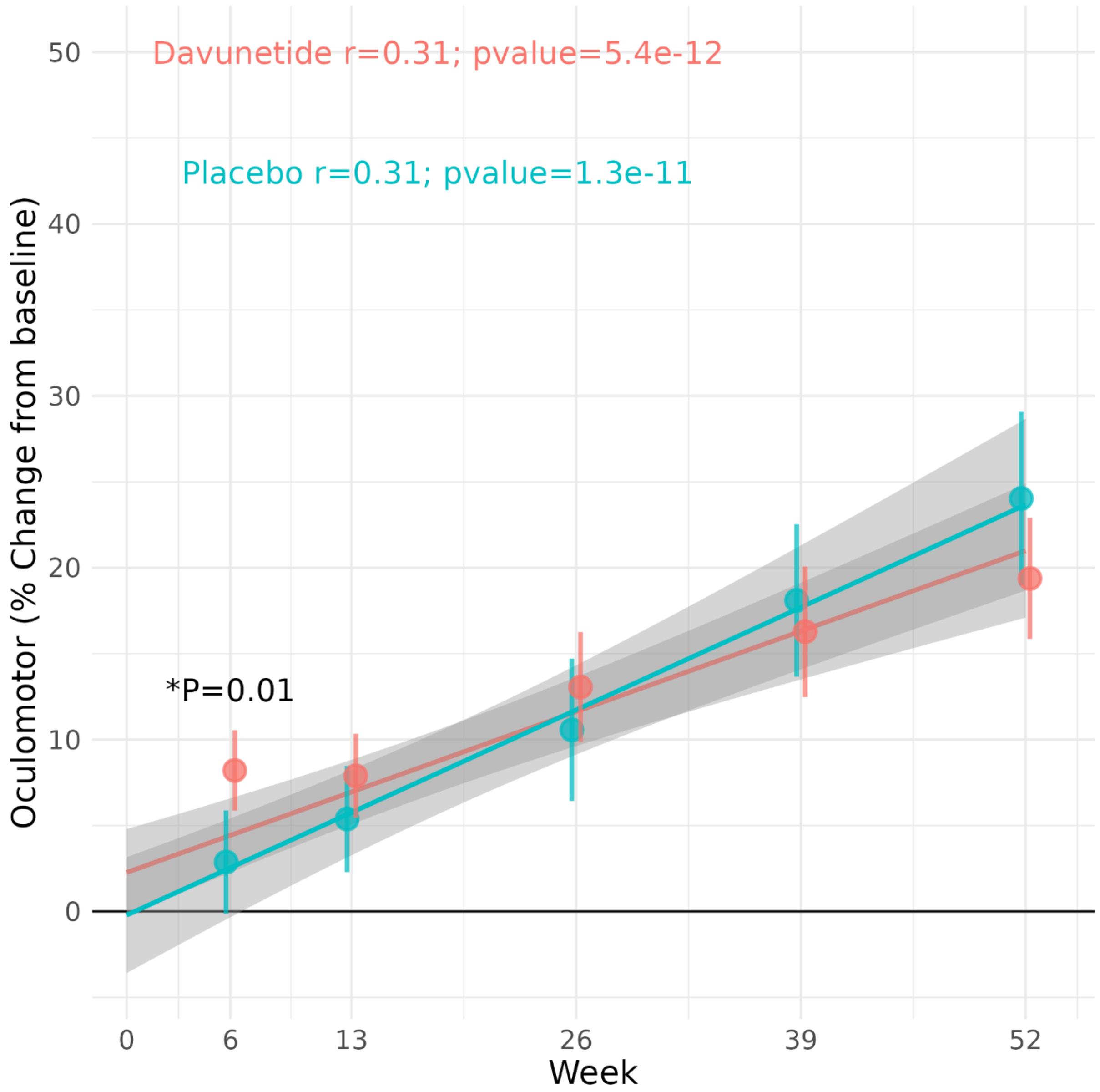

Davunetide r=0.31; pvalue=5.4e-12
Placebo r=0.31; pvalue=1.3e-11
*P=0.01
Oculomotor (% Change from baseline)
50
40
30
20
10
0
0
6
13
26
39
52
Week

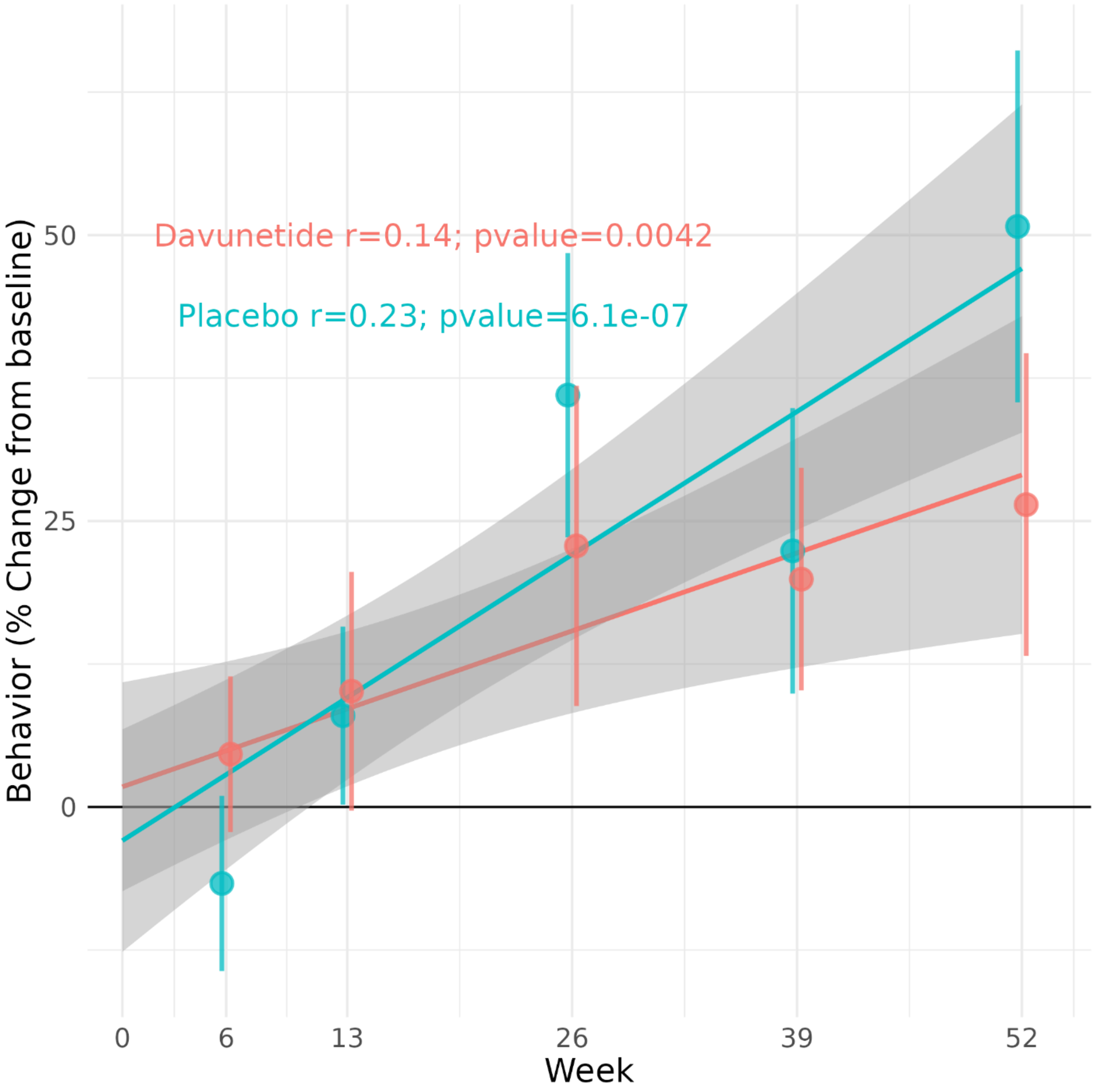
Davunetide r=0.14; pvalue=0.0042
Placebo r=0.23; pvalue=6.1e-07
Behavior (% Change from baseline)
50
25
0
0
6
13
26
39
52
Week

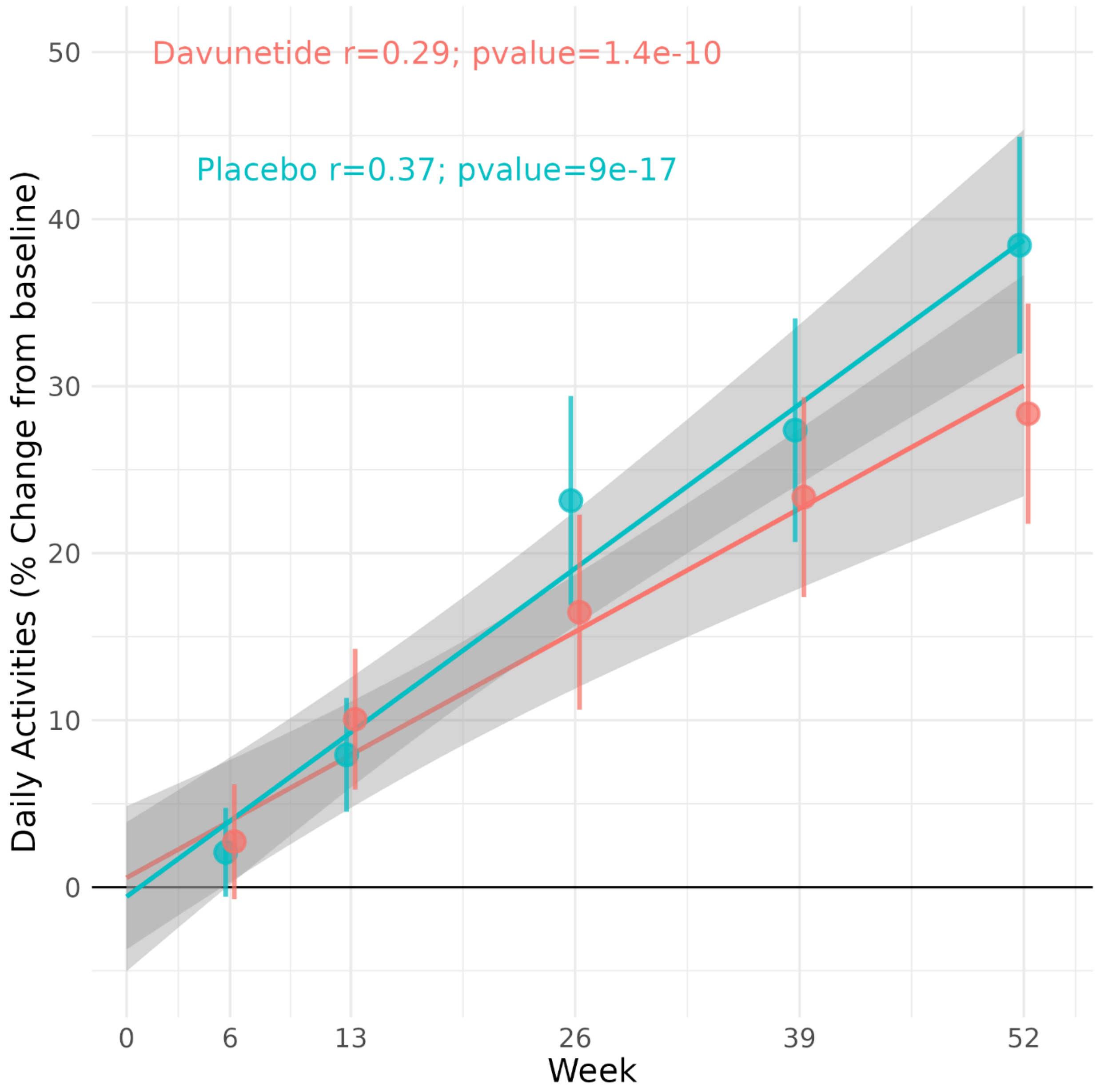
Davunetide r=0.29; pvalue=1.4e-10
Placebo r=0.37; pvalue=9e-17
Daily Activities (% Change from baseline)
50
40
30
20
10
0
0
6
13
26
39
52
Week

References:

1. Boxer AL, Lang AE, Grossman M, Knopman DS, Miller BL, Schneider LS et al. Davunetide in patients with progressive supranuclear palsy: a randomised, double-blind, placebo-controlled phase 2/3 trial. The Lancet Neurology 2014; 13(7): 676-685.
